# Measuring the impact of malaria infection on indicators of iron and vitamin A status: a systematic literature review and meta-analysis

**DOI:** 10.1017/S0007114522000757

**Published:** 2023-01-14

**Authors:** Fanny Sandalinas, Suzanne Filteau, Edward J. M. Joy, Lucia Segovia de la Revilla, Amy MacDougall, Heidi Hopkins

**Affiliations:** 1 Faculty of Epidemiology and Population Health, London School of Hygiene and Tropical Medicine, London, UK; 2 Faculty of Infectious and Tropical Diseases, London School of Hygiene & Tropical Medicine, London, UK

**Keywords:** ferritin, iron, retinol, vitamin A, malaria

## Abstract

Inflammation and infections such as malaria affect estimates of micronutrient status. Medline, Embase, Web of Science, Scopus and the Cochrane library were searched to identify studies reporting mean concentrations of ferritin, hepcidin, retinol or retinol binding protein in individuals with asymptomatic or clinical malaria and healthy controls. Study quality was assessed using the US National Institute of Health tool. Random effects meta-analyses were used to generate summary mean differences. In total, forty-four studies were included. Mean ferritin concentrations were elevated by: 28·2 µg/l (95 % CI 15·6, 40·9) in children with asymptomatic malaria; 28·5 µg/l (95 % CI 8·1, 48·8) in adults with asymptomatic malaria; and 366 µg/l (95 % CI 162, 570) in children with clinical malaria compared with individuals without malaria infection. Mean hepcidin concentrations were elevated by 1·52 nmol/l (95 % CI 0·92, 2·11) in children with asymptomatic malaria. Mean retinol concentrations were reduced by: 0·11 µmol/l (95 % CI −0·22, −0·01) in children with asymptomatic malaria; 0·43 µmol/l (95 % CI −0·71, −0·16) in children with clinical malaria and 0·73 µmol/l (95 % CI −1·11, −0·36) in adults with clinical malaria. Most of these results were stable in sensitivity analyses. In children with clinical malaria and pregnant women, difference in ferritin concentrations were greater in areas with higher transmission intensity. We conclude that biomarkers of iron and vitamin A status should be statistically adjusted for malaria and the severity of infection. Several studies analysing asymptomatic infections reported elevated ferritin concentrations without noticeable elevation of inflammation markers, indicating a need to adjust for malaria status in addition to inflammation adjustments.

Micronutrient deficiencies are a major public health burden, especially in low-income countries, and accurate prevalence estimates are important to guide planning and monitoring of nutritional interventions^(1)^. However, prevalence of micronutrient deficiencies can be incorrectly estimated because certain micronutrient biomarkers are affected by inflammation and infections such as malaria^(2)^. Inflammation is characterised by the acute-phase response to infection, injury or environmental insults. Some acute-phase proteins are also micronutrient markers; for example, serum ferritin, the primary iron storage protein, is a positive acute-phase protein – that is, its concentration increases in response to inflammation – and retinol binding protein (RBP) is a negative acute-phase protein – that is, its concentration decreases in response to inflammation^(2,3)^. Whilst in the absence of inflammation, the concentration of plasma or serum ferritin is positively correlated with the size of the total body iron stores, during inflammation plasma/serum ferritin is raised and does not represent iron stores^(2)^. Infants and young children, as well as women of reproductive age, are at high risk of micronutrient deficiencies due to increased physiological needs^(4)^. They are also at considerably greater risk of contracting malaria, and developing severe disease, than other demographic groups^(5)^. According to the WHO, there were 229 million cases of malaria in 2019^(5)^. More than 90 % of these cases were located in the WHO African region. The presence of parasites can produce a chronic or mild acute-phase response^(6)^. In settings of higher and more holoendemic malaria transmission, more individuals in a population, especially non-pregnant adults, will have some degree of immunity to malaria. Asymptomatic malaria, that is, the presence of parasitaemia in the absence of fever or other malaria-related symptoms, is very common in malaria endemic areas, with some prevalence rates exceeding 50 %^(7)^. There are five well-established malaria parasite species that infect humans, namely *Plasmodium falciparum, P. vivax, P. ovale*, *P. malariae and P. knowlesi. P. falciparum* accounts for 99·7 % of infections in sub-Saharan Africa, while *P. vivax* accounts for 75 % of infections in the Americas^(8)^. Currently used diagnostic methods include microscopy which visualises parasites in stained blood smears, rapid diagnostic tests that detect parasite antigen/s in blood samples;and PCR which identifies the presence of specific malaria genes in a blood sample.

Recently, the WHO published an updated guide on the use of ferritin to assess iron status and the recommended adjustments for inflammation, measured on the basis of C-reactive protein (CRP) and alpha-1-acid glycoprotein (AGP) concentrations in blood serum/plasma^(9)^. Ferritin values may differ by malaria infection status^(10)^ after correcting for inflammation defined by raised CRP and/or AGP, and the updated WHO guidelines mention malaria as a possible factor for adjustment. Other biomarkers are also likely to be affected by malaria. Retinol, the predominant circulating form of vitamin A in the blood, is known to be affected by malaria infection^(11)^. In children aged 6–59 months in Ghana, increasing malaria parasite density was significantly associated with decreasing serum retinol concentrations^(11)^. These reductions have been attributed largely to the inflammatory response. As the measurement of serum retinol requires expensive laboratory equipment, some micronutrient surveys measure its carrier protein, RBP, instead of retinol itself. In young children in Liberia, Larson *et al.* found a significant added effect of malaria on RBP concentrations and vitamin A deficiency prevalence estimates even after adjusting for CRP and AGP using the regression approach^(12)^. There is also a growing interest in the impact of malaria on hepcidin, the iron regulatory hormone^(13,14)^. Hepcidin seems to be upregulated in malaria infection even in asymptomatic human infection^(7)^. This results in a blockage of iron absorption from the diet and a redistribution of iron into the body, away from the serum.

Taking into account the effect of malaria on micronutrient biomarkers has the potential to significantly modify the estimation of the prevalence of micronutrient deficiencies derived from large population-based surveys such as national micronutrient surveys. This research estimated the effect of malaria on several biomarker values (ferritin, hepcidin, retinol and RBP) by performing a meta-analysis of studies comparing biomarker values in individuals infected with malaria and individuals without malaria infection.

## Methods

The protocol of this systematic review has been published on PROSPERO on the 24 September 2021: CRD42021279974. Ethical approval was not needed as the data used in the analysis are fully available in the public domain. We followed the Meta-analysis of Observational Studies in Epidemiology (MOOSE) reporting checklist for this systematic review and meta-analysis.

### Eligibility criteria

Randomised controlled trials or quasi-randomised controlled trials, prospective observational studies with data collection at multiple time points and cross-sectional studies with a control group that measured selected biomarkers in malaria-infected individuals were eligible. In children and non-pregnant adults, studies that distinguished asymptomatic and clinical malaria cases were included; studies in these populations that combined asymptomatic and symptomatic infections in a single malaria group were excluded. Studies that provided an intervention believed to impact the iron or vitamin A status of the participants were only included if data from a control group, or baseline data, could be extracted. Studies of human participants of any age and sex were eligible. As individuals suffering from severe malaria are often not sampled in large population-based surveys that measure micronutrient status, we decided not to include reports that only recruited participants with severe malaria. However, if individuals with severe malaria were included in papers that meet other selection criteria, they were analysed separately. For similar reasons, studies that recruited individuals based on their being anaemic or having another disease that is likely to affect iron or vitamin A metabolism (sickle cell disease and thalassemia) were not included.

### Search strategy

**Fig. 1.**
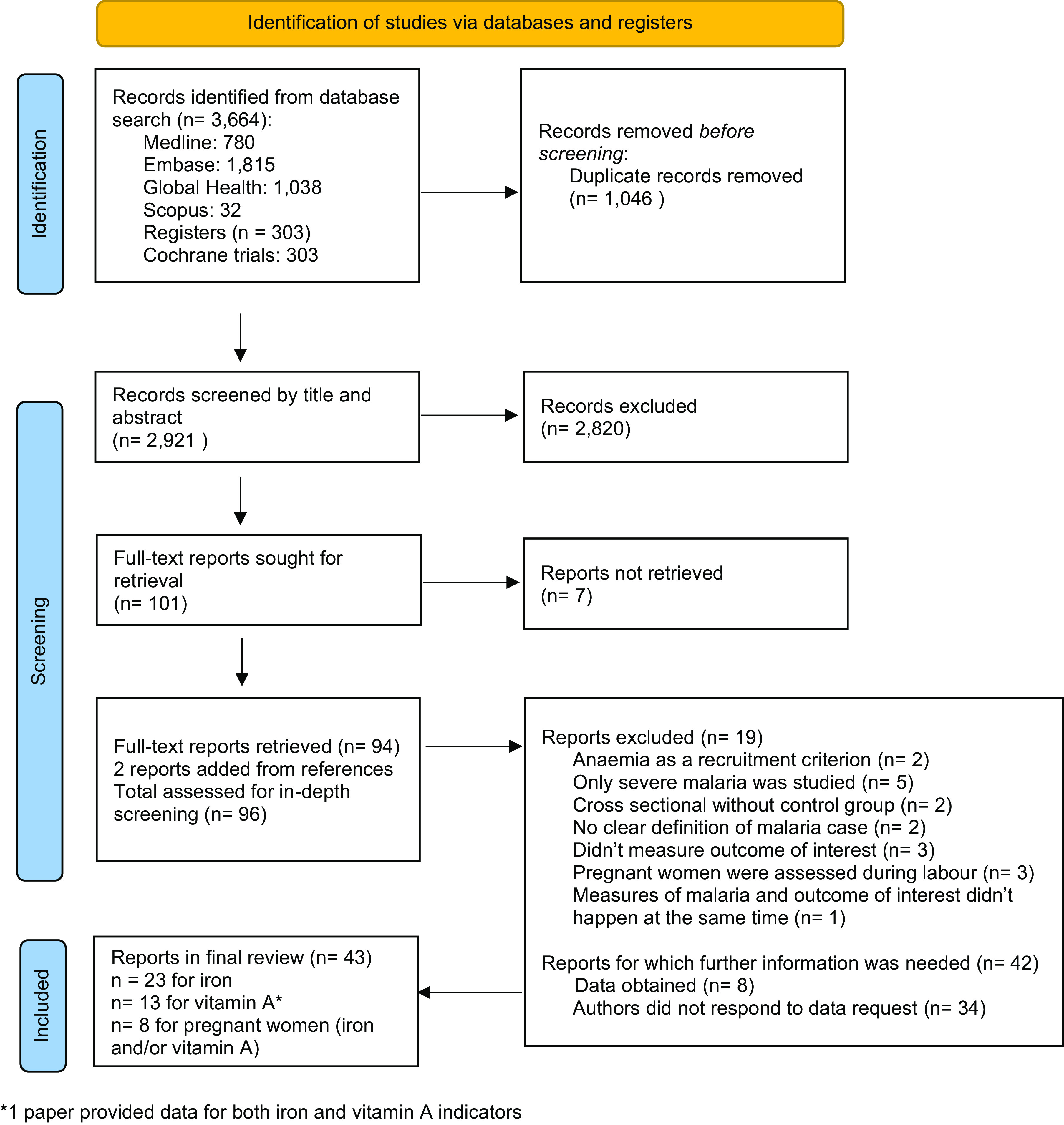
Preferred Reporting Items for Systematic Reviews and Meta-Analysis (PRISMA) flow diagram of publications screened in a systematic review of the impact of malaria infection on indicators of iron and vitamin A status.

Medline, Embase, Web of Science, Scopus and the Cochrane library were searched in April 2021. The search strategy included the use of Medical Subject Heading (MeSH) terms and text words, with the use of explosion technique. The complete search strategy, which was reviewed by a qualified librarian, is included in supplementary file 1. There was no restriction on the date of publication. Reports written in English, French and Spanish were eligible. Abstracts and unpublished studies were not considered. Two reviewers, FS and LSR, screened each record independently, in a two-stage process: first the reviewers examined titles and abstracts. The full texts were then retrieved and the reviewers examined the full-text reports for compliance with the eligibility criteria. After retrieval of articles from the search, the reference lists of all selected articles were checked for other potentially relevant articles; two additional papers were identified. Disagreements were discussed between the two reviewers until an agreement could be reached. References were managed in EndNote 20 (Clarivate Analytics). The Preferred Reporting Items for Systematic Reviews and Meta-Analysis (PRISMA) flow chart of identified studies is illustrated in Fig. 1.

### Data extraction and statistical analysis

Data were extracted into MS Excel by FS using a tool which included author name, study design, sample size, population group, age group, year when the study took place, country, malaria endemicity profile, method of diagnosis of malaria, clinical definition of malaria, malaria species, and the summary statistics of ferritin, hepcidin, retinol and RBP. Although Hb and soluble transferrin receptors are sometimes used to describe iron status, we did not include them in our analysis, as Hb is not a specific indicator of iron deficiency and soluble transferrin receptors concentrations are not reported as often as ferritin concentrations. The malaria endemicity profile was defined by the *Plasmodium falciparum* prevalence rate (PfPR) among children aged 2–10 years, as described in the Malaria Atlas Project^(15)^. The different categories were defined by the WHO^(16)^: PfPR < 1 %: very low intensity, PfPR ≥ 1 % and < 10 %: low intensity, PfPR ≥ 10 and < 35 %: moderate intensity, PfPR ≥ 35 %: high intensity. The data from three clinical groups were included: healthy participants with negative malaria test results, asymptomatic participants with positive malaria test and without clinical sign of illness, and a clinical malaria group who had a positive malaria test and fever. For prospective studies, the biomarker measurement at admission was considered the measurement of the malaria group, and the measure at the final follow-up point was considered the measurement of the control group. We analysed separately three population groups: children, non-pregnant adults and pregnant women. When a study provided data for different malaria species, or for different age groups, the corresponding data were entered into different datasets to allow for subgroup analysis, which explains why there is a greater number of datasets than studies. If the data from the same group were used for two comparisons in the same meta-analysis, we halved the number of participants from this group in each comparison, following the method described in the Cochrane Handbook^(17)^. Authors were contacted if the relevant information was not available in the report. The risk of bias of all included studies was assessed by FS, using the US National Institute of Health quality assessment tool for observational cohort and cross-sectional studies. This tool contains fourteen questions around key concepts for evaluating the internal validity of a study. They are not intended to create a final score but help to assess potential selection, information, measurement and confounding biases. The use of this tool for cross-sectional studies was recommended in a recent review by Ma *et al.* ^(18)^. For the outcomes specified earlier, we reported the mean value of different groups (healthy control group, asymptomatic malaria group and clinical malaria group), as well as the 95 % CI or the standard deviation. We calculated the mean difference with 95 % CI between groups. We also attempted to calculate missing information from other reported measurements, if possible. When the geometric mean was provided, we transformed it into an arithmetic mean using the method explained by Higgins^(19)^. We generated meta-analyses based on the severity of the disease, either asymptomatic or clinical malaria and specific outcomes, such as ferritin or retinol concentrations. We first calculated a summary statistic for each study to describe the observed malaria effect. As our data were continuous, the summary statistic was a difference between means and a 95 % CI. When the data came from prospective studies, we followed the method described in the Cochrane Handbook to impute a standard deviation change from baseline to endline^(17)^. We then used a random effects meta-analysis for combining data, as we anticipated that there may be natural heterogeneity between studies attributable to the different populations and settings. The study weights were equal to the inverse of the variance of each study’s effect estimate according to the methodology developed by DerSimonian and Laird^(20)^. We generated forest plots and we provided a CI, which communicates the precision (or uncertainty) of the summary estimate and a *P*-value.

When data were available, and when more than one study provided relevant data for meta-analysis, we conducted the following subgroup analyses: species of malaria (falciparum *v*. vivax), malaria endemicity profile of the country (low *v*. moderate *v*. high intensity of transmission, as defined by^(15,16)^), method of diagnosis of malaria (rapid diagnostic tests *v*. microscopy or both), age of children (under *v*. over 5 years old) and design of the study (cross-sectional *v*. prospective study). Heterogeneity was assessed using the I^2^ statistic. Both a qualitative (funnel plot) and a quantitative (Egger’s regression test) approach were used to examine potential publication biases. An influence analysis was conducted to determine the effect of removing each included study on the overall effect and 95 % CI using the technique described by Viechtbauer and Cheung^(21)^. The meta-analyses, funnel plot, Egger’s regression test and influence analysis were conducted using RStudio software version 1.3.959^(22)^ with the dmetar (v. 0.0.9000^(23)^) and meta-packages^(24)^. The workbooks for meta-analysis (version 1.5) developed by Suurmond were used to perform the subgroup analyses^(25)^. A *P*-value of < 0·05 for meta-analyses was considered statistically significant.

## Results

Of 101 full-text reports screened, 43 papers describing 44 studies conducted in 27 countries met selection criteria^(7,10,11,13,26–65)^ (Fig. 1). Twenty-nine studies were conducted in fifteen African countries^(10,11,13,26,28–31,38–41,46,47,49–53,55,57,62,63)^, nine were conducted in Asia^(27,32,36,37,42,44,48,56,58)^, two in Oceania^(54,64)^, three in Europe^(43,45,48)^(two studied imported cases and one experimental malaria infection) and one in the Americas^(35)^ (Table 1). Among the included studies, twenty-three reported on ferritin and/or hepcidin concentrations in adults and/or children^(7,10,13,45–64)^, thirteen reported on retinol or RBP concentrations in adults and/or children^(11,34–44,63)^, and eight reported on either ferritin, hepcidin, retinol or RBP concentrations in pregnant women^(26–33)^. Eight studies compared the ferritin or retinol level between groups with different severity of malaria^(13,30,40,51,52,54,56,64)^. Thirty-four studies were cross-sectional^(11,13,26–34,36–40,42,44,50–55,57–60,62–64)^, whereas ten were prospective^(7,41,43,45–49,56,61)^. The predominant species of malaria was *P. falciparum* in forty-one studies^(7,11,13,26,28–34,36–43,45–57,59,60,62–64)^, whereas *P. vivax* was either predominant or as present as *P. falciparum* in four studies^(27,35,44,58)^. A broad range of endemic profiles were represented. The pooled sample size for the analysis of children (*N* 14 330) was larger than for adults (*N* 985) (Table 2). The risk of bias assessment revealed that the majority of studies had a low or unclear risk of bias (online Supplementary Table 1).

**Table 1.** Characteristics of the studies included in the systematic review of the impact of malaria infection on indicators of iron and vitamin a status

*n*	Author, publication year	Period the study was conducted	Country	Study design	Study participants	Malaria endemicity profile*	Total cases	Clinical severity	Malaria species	Diagnostic of Malaria	Outcomes measured
Studies reporting on iron status indicators											
1	Barffour *et al.* 2018^(10)^	2012–2013	Zambia	CS nested in a cluster-RCT	Children 4–8 years	Moderate intensity, seasonal transmission; two cross-sectional surveys conducted, one during high and one in low transmission period	744	A and control	F	RDT (HRP2) and P†	Ferritin
2	Beesley *et al.* 2000^(45)^	NA	Europe	Prospective study	Adults	Imported cases	36	C	F	P	Ferritin
3	Burte *et al.* 2013^(13)^	2008–2010	Nigeria	CS as part of a larger prospective study	Children 6 months–12 years	High intensity, year-round	117	DC, C, CM, SMA and control	F	P	Ferritin
4	Castberg *et al.* 2018^(46)^	2014–2015	Ghana	Prospective study as part of a larger study	Children 1–12 years	Moderate intensity, year-round	98	C	F	P	Ferritin, hepcidin
5	Cercamondi *et al.* 2010^(47)^	NA	Benin	Prospective study	Adults (women)	High intensity, seasonal transmission; survey conducted during high transmission season	23	A	F	P	Ferritin, hepcidin
6	de Mast *et al.* 2010^(7)^	NA	Indonesia	Prospective study	Children 5–15 years	Low intensity, year-round	108	A and control	F and V	P	Ferritin, hepcidin
7	de Mast *et al.* 2009^(48)^	1999–2003	Europe	Prospective experimental human malaria infection	Adults	Experimental infection	5	C and control	F	P	Ferritin, hepcidin
8	Diallo *et al.* 2020^(26,65)^	2011–2014	Burkina Faso	CS surveys included in a prospective study	Pregnant women and infants	High intensity, year-round	Women: 916	Positive malaria test and control	F	P	Ferritin
9	Dreyfuss *et al.* 2000^(27)^	1994–1997	Nepal	CS as part of an RCT	Pregnant women	Very low intensity, year-round	288	C and control	V	P	Ferritin
10	Glinz *et al.* 2015^(49)^	NA	Cote d’Ivoire	Prospective study	Children 11–17 years	Moderate intensity, year-round	17	A	F	P	Ferritin, hepcidin
11	Jeremiah *et al.* 2007^(50)^	2005–2006	Nigeria	CS	Children 1–8 years	High intensity, year-round	240	A and control	F	P	Ferritin
12	Kabore *et al.* 2020^(51)^	2016–2017	Burkina Faso	CS	Children (over 3 months) and adults	Moderate intensity, seasonal transmission; survey conducted during high transmission season	Children: 722 Adults: 396	A and C and control	F	P	Ferritin, hepcidin
13	Kivibidila *et al.* 1999^(52)^	1991	DR Congo	CS	Children 6 months–16 years	Moderate intensity	44	CM, C and control	F	P	Ferritin
14	Mockenhaupt *et al.* 2000^(28)^	1998	Ghana	CS	Pregnant women	High intensity	530	Positive malaria test and control	F and one case of P. Ovale	P and PCR	Ferritin
15–18	Muriuki *et al.* 2020^(53,73–76)^	2013	Burkina Faso	CS	Children 12 months	High intensity, year-round	348	A and control	F	P	Ferritin, hepcidin
		2001	The Gambia	CS as part of a prospective study	Children 2–6 years	Moderate intensity, seasonal transmission; survey conducted during high transmission season	753	A and control	F	P	Ferritin, hepcidin
		2012–2014	Kenya	CS as part of a prospective study	Children under 5 years	Low intensity, seasonal transmission; survey conducted during high transmission season	1484	A and control	F	P	Ferritin, hepcidin
		2003–2005	Uganda	CS as part of an RCT	Children under 5 years	Moderate intensity, year-round	1374	A and control	F	P	Ferritin, hepcidin
19	Mwangi^(29)^	2011–2014	Kenya	CS as part of an RCT	Pregnant women	Low intensity, year-round	470	C and control	F	P	Ferritin
20	O’Donnell *et al.* 2009^(54)^	1993– 1996	Papua New Guinea	CS	Children under 4 years	Low intensity	495	C, severe malaria and control	F	P	Ferritin
21	Odunukwe *et al.* 2000^(55)^	NA	Nigeria	CS	Adults	High intensity, year-round	300	A and control	F and malariae	P	Ferritin
22	Phillips *et al.* 1986^(56)^	NA	Thailand	Prospective study	Adults	Very low intensity	23	C, CM and control	F	P	Ferritin
23	Righetti *et al.* 2013^(57)^	2010	Cote d’Ivoire	CS as part of prospective study	Children 6 months–8 years and adults	High intensity, year-round	Children: 246 Adults: 92	A and C	F	RDT (HRP2) and P‡	Ferritin
24	Saad *et al.* 2012^(30)^	2010	Sudan	CS	Pregnant women	Low intensity	64	Severe, C and control	F	P	Ferritin
25	Seyrek *et al.* 2004^(58)^	NA	Turkey	CS	Adults	Very low intensity	31	C and control	V	P	Ferritin
26	Shulman *et al.* 1996^(31)^	1993	Kenya	CS	Pregnant women	Low intensity, seasonal. Survey conducted during a low transmission period	275	C and control	F	P	Ferritin
27	Stoltzfus *et al.* 1997^(59)^	1994	Zanzibar	CS	Schoolchildren 5–19 years	Moderate intensity, year-round	3605	A and control	F	P	Ferritin
28	Stoltzfus *et al.* 2000^(60)^	1996	Zanzibar	CS	Children under 5 years	Moderate intensity, year-round	464	A and control	F	P	Ferritin
29	Uscategui *et al.* 2009^(61)^	2002	Colombia	Prospective case study	Children 4–10 years	Very low intensity	89	C	F and V	P	Ferritin
30	Verhoef *et al.* 2001^(62)^	1997	Kenya	CS	Children 2–36 months	Moderate intensity, seasonal transmission; survey conducted during high transmission season	318	A and control	F	P	Ferritin
31	Wessells *et al.* 2014^(63)^	2009	Burkina Faso	CS data from an RCT	Children 6–23 months	High intensity, year-round	437	A and control	F	ELISA (HRP2)	Ferritin
32	Wessells *et al.* 2017^(33)^	2014–2015	Niger	CS as part of an RCT	Pregnant women	Moderate intensity	787	Positive malaria test and control	F	ELISA (HRP2)	Ferritin
33	Williams *et al.* 1999^(64)^	1994	Vanuatu	CS	Children, mean age: 11 years	Low intensity, seasonal transmission; survey conducted during high transmission season	115	A, C and control	F and V	P	Ferritin
Studies reporting on vitamin A status indicators											
1	Barffour *et al.* 2018^(34)^ §	2012–2013	Zambia	CS nested in a cluster RCT	Children 4–8 years	Moderate intensity, seasonal transmission; two cross-sectional surveys conducted, one during high and one in low transmission period	744	A and control	F	RDT (HRP2) and P†	Retinol
2	Carmona *et al.* 2008^(35)^ ||	2002	Colombia	Prospective case study	Children 4–10 years	Very low intensity	89	C	F and V	P	Retinol
3	Das *et al.* 1996^(36)^	1992–1993	India	CS	Children 2–11 years	Low intensity	173	A, C and severe malaria	F	P	RBP, retinol
4	Davis *et al.* 1993^(37)^	1991	China	CS	Adults	Very low intensity	27	C and control	F and V	P	Retinol
5	Diatta *et al.* 2013^(38)^	NA	Senegal	CS	Children under 5 years	Low intensity, seasonal transmission; survey conducted during high transmission season	312	A and control	F	P	Retinol
6	Filteau *et al.* 1993^(11)^	1990–1991	Ghana	CS as part of a randomised double-blind study	Children 6–59 months	High intensity, year-round	59	A and control	F	P	Retinol
7	Inocent *et al.* 2007^(39)^	2004–2005	Cameroon	CS	Children 0–6 years	High intensity	116	C and control	F	P	Retinol
8	Mfonkeu *et al.* 2010^(40)^	2007	Cameroon	CS	Children 6 months–14 years	High intensity	139	C, MA, CM, CM & MA and control	F	P	Retinol
9	Nussenblatt *et al.* 2002^(41)^	1998	Uganda	Prospective study	Children 1–10 years	High intensity, year-round	273	C	F	P	Retinol
10	Raza *et al.* 2009^(42)^	2006–2007	India	CS	Children 2–5 years	Low intensity	170	C and control	F	P	Retinol
11	Stuetz *et al.* 2005^(32)^	1998–2000	Thailand	Secondary samples analysis	Pregnant women	Low intensity, year-round	108	Positive malaria test and control	F	P	Retinol
12	Tabone *et al.* 1992^(43)^	NA	Europe	Prospective study	Adults	Imported cases	7	C	F	P	Retinol, RBP
13	Thurnman *et al.* 1991^(44)^	NA	Thailand	CS	Adults	Very low intensity	45	C and control	F, V and mixed	P	Retinol
14	Wessells *et al.* 2014^(63)^	2009	Burkina Faso	CS data from an RCT	Children 6–23 months	High intensity, year-round	437	A and control	F	ELISA (HRP2)	RBP

CS, cross-sectional; A, asymptomatic malaria; F, falciparum; RDT, rapid diagnostic test; HRP2, histidine-rich protein 2; NA, non-available; C, clinical-uncomplicated malaria; P, parasitaemia by microscopy; DC, disease control; CM, cerebral malaria; SMA, severe malarial anaemia; V, vivax; RCT, randomised controlled trial; MA, malarial anaemia; RBP, retinol binding protein. ‘Control’ is defined as healthy children with no malaria detected by the study-specific diagnostic test.

* Seasonality was defined by the author and the information was not systematically reported. The intensity of transmission was defined by the *Plasmodium falciparum* prevalence rate (PfPR) among children aged 2–10 years, as described in the Malaria Atlas Project^(15)^. As defined by WHO^(16)^, PfPR < 1 %: very low intensity, PfPR ≥ 1 % and < 10 %: low intensity, PfPR ≥ 10 and < 35 %: moderate intensity, and PfPR ≥ 35 %: high intensity. The PfPR is the proportion of the population found to carry asexual blood-stage parasitaemia, a basis for the classical categorical measures of malaria transmission.

† Malaria parasitaemia was detected by RDT and/or microscopy.

‡ Malaria diagnosis was by microscopy-confirmed RDT.

§ Participants are the same as in ref. 10.

|| Participants are the same as in ref. 61.

**Table 2.** Characteristics of individuals and settings from studies included in the systematic review of the impact of malaria infection on indicators of iron and vitamin A status (Number and percentages)

	Children		Non-pregnant adults		Pregnant women		Total	
	*n*	%	*n*	%	*n*	%	*n*	%
Number of studies included	27		11		8		46*	
Number of participants in the included studies	14 330		985		3438		18 753	
Age in years (mean ± sd) of participants in the included studies								
Mean	6·2		29·6		24·1			
sd	4		9		5·1			
Estimated malaria transmission intensity during the survey in the included studies†								
Very low (< 1 %)	2	7 %	4	37 %	1	13 %	7	16 %
Low (1–10 %)	7	26 %	0		4	50 %	11	14 %
Moderate (10–35 %)	8	30 %	1	9 %	1	13 %	10	23 %
High (> 35 %)	10	37 %	3	27 %	2	25 %	15	34 %
Imported cases treated in Europe or experimental infection	0		3	27 %	0		3	6 %
Seasonality of transmission in the included studies								
Holoendemic	12	44 %	2	18 %	4	40 %	18	40 %
Seasonal	6	22 %	2	18 %	1	20 %	9	19 %
Imported cases treated in Europe or experimental infection	0		3	28 %	0		3	7 %
Not specified by authors	9	34 %	4	36 %	2	40 %	15	34 %

* Two reports provided data for children and adults.

† As defined in ref. 15 and 16.

### Asymptomatic malaria and ferritin concentrations in children and adults

**Fig. 2.**
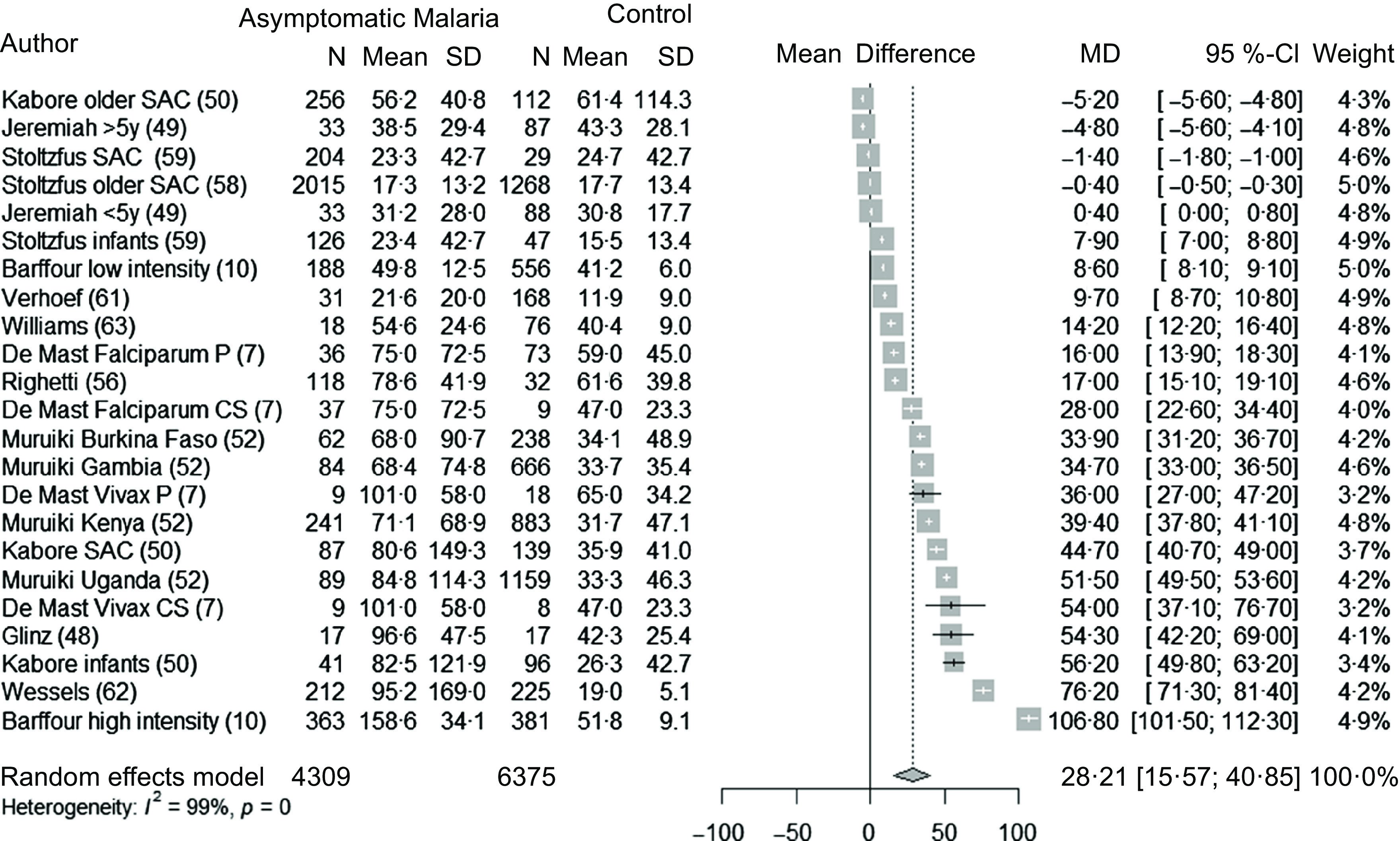
Forest plot of differences in ferritin concentrations (µg/l) between children with asymptomatic malaria and control group, using the random effect model. The grey squares represent the mean difference from each study, while the horizontal line represents the corresponding 95 % CI. The hollow diamond represents the overall pooled effects, while the left and right points of the diamond represent the corresponding 95 % CI. SAC, school age children; P, prospective; CS, cross-sectional; MD, mean difference.

Fifteen studies^(7,10,49–51,53,57,59,60,63,64)^ (twenty-three datasets) analysed the association between malaria and ferritin concentrations in asymptomatic children (4309 children with malaria infection and 6375 control children). Overall, ferritin concentrations were 28·2 µg/l (95 % CI 15·6, 40·9, *P* < 0·001) greater in children with asymptomatic malaria compared with control groups (Fig. 2). The subgroup analyses did not reveal any differences (Table 3). There was strong evidence of between-study heterogeneity of effect (I^2^ = 99 %). Heterogeneity was not explained by descriptive study factors (Table 3). The sensitivity analysis showed the stability of the pooled results after the leave-one-out analysis (Supplementary Fig. 1), and all studies had a low or unclear risk of bias (Table 4). The funnel plot and the Egger test did not show significant asymmetry, indicating no significant publication bias for this analysis (Supplementary Fig. 2).

**Table 3.** Results of subgroup analyses for ferritin concentration (µg/l) in children with asymptomatic malaria parasitaemia and control group (Mean difference and 95 % confidence interval)

	Number of datasets	Mean difference	95 % CI	*I* ^2^ for heterogeneity	*P*_subgroup_
Endemic profile					0·69
High intensity	5	24·1	–4·34, 52·4	99 %	
Moderate intensity	12	28·6	9·8, 47·4	99 %	
Low intensity	6	30	18·4, 41·7	99 %	
Malaria species					0·06
* P. falciparum*	21	26·6	14·2, 39	99 %	
* P. vivax*	2	43·3	25·5, 61·1	66 %	
Age					0·12
< 60 months	12	30·6	16·8, 44·3	99 %	
> 60 months	11	23·3	3·4, 43·2	99 %	
Diagnostic method					0·51
Parasitaemia by microscopy	20	25·1	14·5, 35·7	99 %	
RDT or microscopy	2	57·7	–38·7, 154	99 %	
Design of the study					0·50
Cross-sectional	20	26·9	13·9, 39·9	99 %	
Prospective	3	34·7	12·9, 56·6	96 %	

RDT, rapid diagnostic test detecting histidine-rich protein 2.

**Table 4.** Summary of meta-analyses results by biomarker in children and adults with malaria parasitaemia compared with control group (Mean difference and 95 % confidence interval)

	All studies					Only studies with low or unclear risk of bias				
	Number of datasets	Mean difference	95 % CI	I^2^ for heterogeneity	*P*	Number of datasets	Mean difference	95 % CI	I^2^ for heterogeneity	*P*
Ferritin (µg/l)										
Children										
Asymptomatic malaria	23	28·2	15·6, 40·9	99 %	<0·001	23	28·2	15·6, 40·9	99 %	<0·001
Clinical malaria	9	366	162, 570	91 %	0·003	7	334	106, 563	92 %	0·01
Adults										
Asymptomatic malaria	4	28·5	8·1, 48·9	51 %	0·02	3	20·5	4·8, 36·3	0 %	0·03
Clinical malaria	5	493	–219, 1206	83 %	0·13	2	155	–758, 1069	57 %	0·28
Pregnant women	8	26·8	5·8, 47·7	100 %	0·02	7	18·1	5·6, 30·7	99 %	0·01
Hepcidin (nmol/l)										
Children										
Asymptomatic malaria	12	1·52	0·92, 2·11	96 %	<0·001	12	1·52	0·92, 2·11	96 %	<0·001
Clinical malaria	2	10·8	–18·1, 39·7	0 %	0·13	1	10·6	5·4, 15·8	NA	<0·001
Adults										
Asymptomatic malaria	2	0·3	–10·3, 10·9	52 %	0·78	2	0·3	–10·3, 10·9	52 %	0·78
Clinical malaria	2	6·3	–21·6, 34·3	52 %	0·21	2	6·3	–21·6, 34·3	52 %	0·21
Retinol (µmol/l)										
Children										
Asymptomatic malaria	4	–0·11	–0·22, −0·01	60 %	0·04	3	–0·10	–0·18, −0·03	21 %	0·03
Clinical malaria	6	–0·43	–0·71, −0·16	97 %	0·01	4	–0·36	–0·77, 0·06	93 %	0·07
Adults										
Clinical malaria	5	–0·73	–1·11, −0·36	54 %	0·005	1	–1·03	–1·40, −0·66	NA	<0·001
Pregnant women	1	–0·54	–0·67, −0·41	NA	<0·001	1	–0·54	–0·67, −0·41	NA	<0·001

NA, non-applicable.

**Fig. 3.**
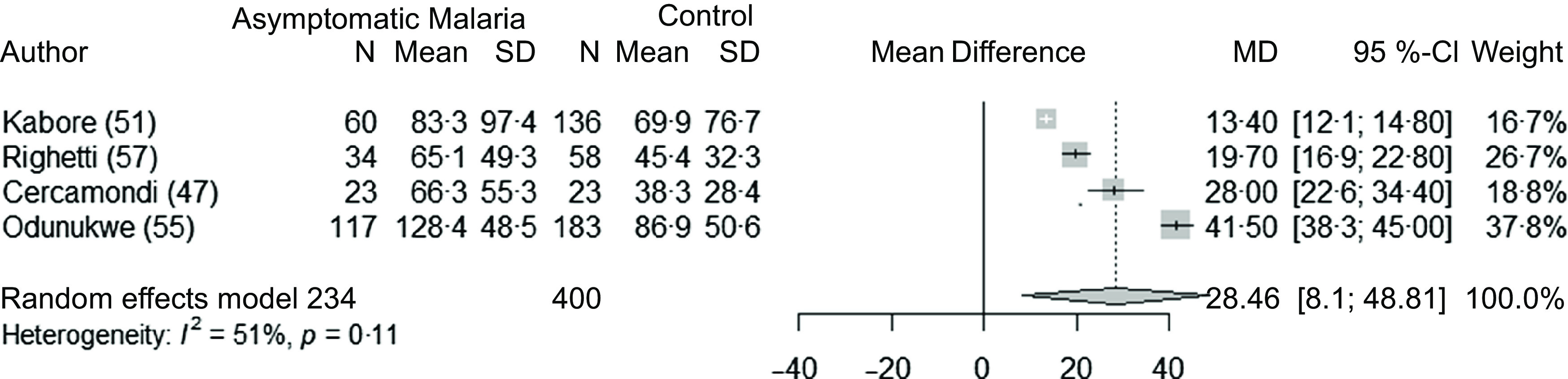
Forest plot for differences in ferritin concentrations (µg/l) between adults with asymptomatic malaria and control group using the random effect model. The grey squares represent the mean difference from each study, while the horizontal line represents the corresponding 95 % CI. The hollow diamond represents the overall pooled effects while the left and right points of the diamond represent the corresponding 95 % CI. MD, mean difference.

A greater ferritin concentration was also observed in non-pregnant adults with asymptomatic malaria (28·5 µg/l, 95 % CI 8·1, 48·8, *P* = 0·02) (Fig. 3). This mean difference was calculated for 234 adults with malaria and 400 control adults, from 4 studies conducted in sub-Saharan Africa in settings with moderate or high intensity of malaria transmission^(47,51,55,57)^. Given the limited number of studies, we did not perform subgroup analyses. The heterogeneity was moderate (51 %) and the sensitivity analysis showed the stability of the pooled result (Supplemental Fig. 3, Supplemental Fig. 4 and Table 4)

### Clinical malaria and ferritin concentrations in children and adults

**Fig. 4.**
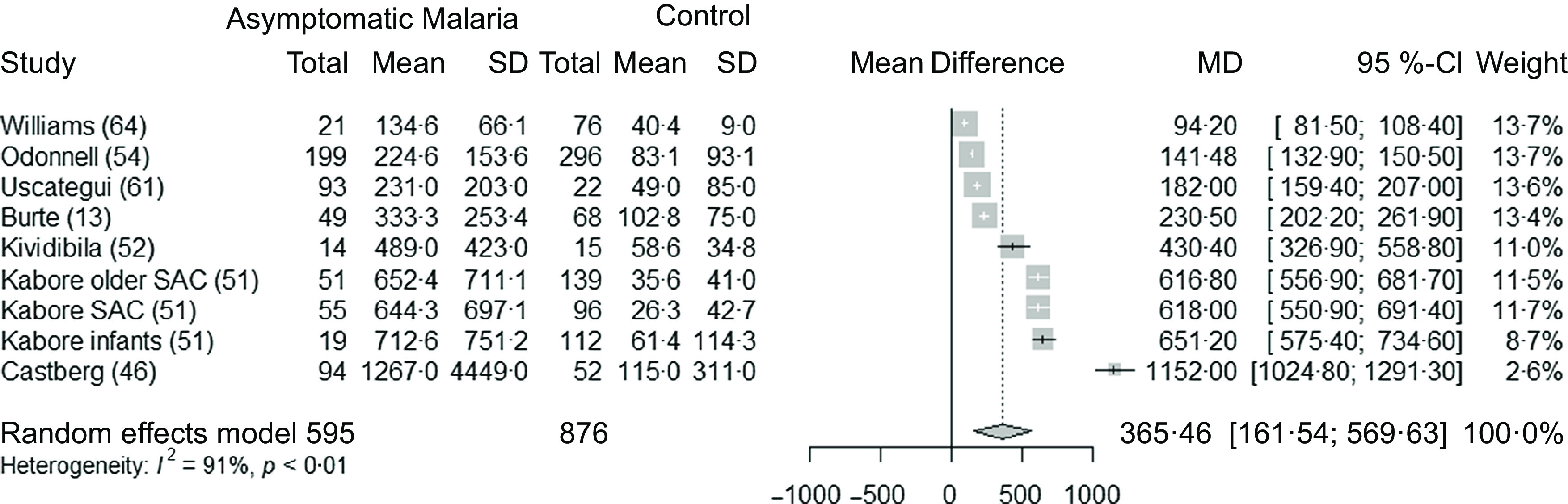
Forest plot for differences in ferritin concentrations (µg/l) in children between clinical malaria and control group using the random effect model. The grey squares represent the mean difference from each study, while the horizontal line represents the corresponding 95 % CI. The hollow diamond represents the overall pooled effects, while the left and right points of the diamond represent the corresponding 95 % CI. SAC, school age children; MD, mean difference.

Seven studies^(13,46,51,52,54,61,64)^ (nine datasets) analysed the association between clinical malaria and ferritin concentrations in children (595 children with clinical malaria and 876 healthy, control children). These studies were conducted in Africa, in Oceania and in the Americas. Overall, ferritin concentrations were 366 µg/l (95 % CI 162, 570) *P* < 0·003) greater in children with clinical malaria compared with control group (Fig. 4). The sub-group analyses showed that the difference in mean ferritin was the greatest in settings with moderate transmission, compared with low transmission (Table 5). The sensitivity analysis showed the stability of the pooled results after the leave-one-out analysis (Supplementary Fig. 5) and after removing the studies with high risk of bias (Table 4). The funnel plot revealed a publication bias for this analysis, with an underreporting of small studies (Supplementary Fig. 6).

**Table 5.** Results of subgroup analyses for ferritin (µg/l) in children with clinical malaria parasitaemia and control group (Mean difference and 95 % confidence interval)

	Number of datasets	Mean difference	95 % CI	I^2^ for heterogeneity	*P*_subgroup_
Endemic profile					<0·001
Moderate intensity	5	688	457, 919	94 %	
Low intensity	3	138	89, 188	96 %	
Age					0·13
<60 months	4	637	233, 1042	99 %	
>60 months	5	304	119, 490	99 %	
Study design					0·57
Cross-sectional	7	389	209, 570	99 %	
Prospective	2	665	–290, 1620	99 %	

**Fig. 5.**
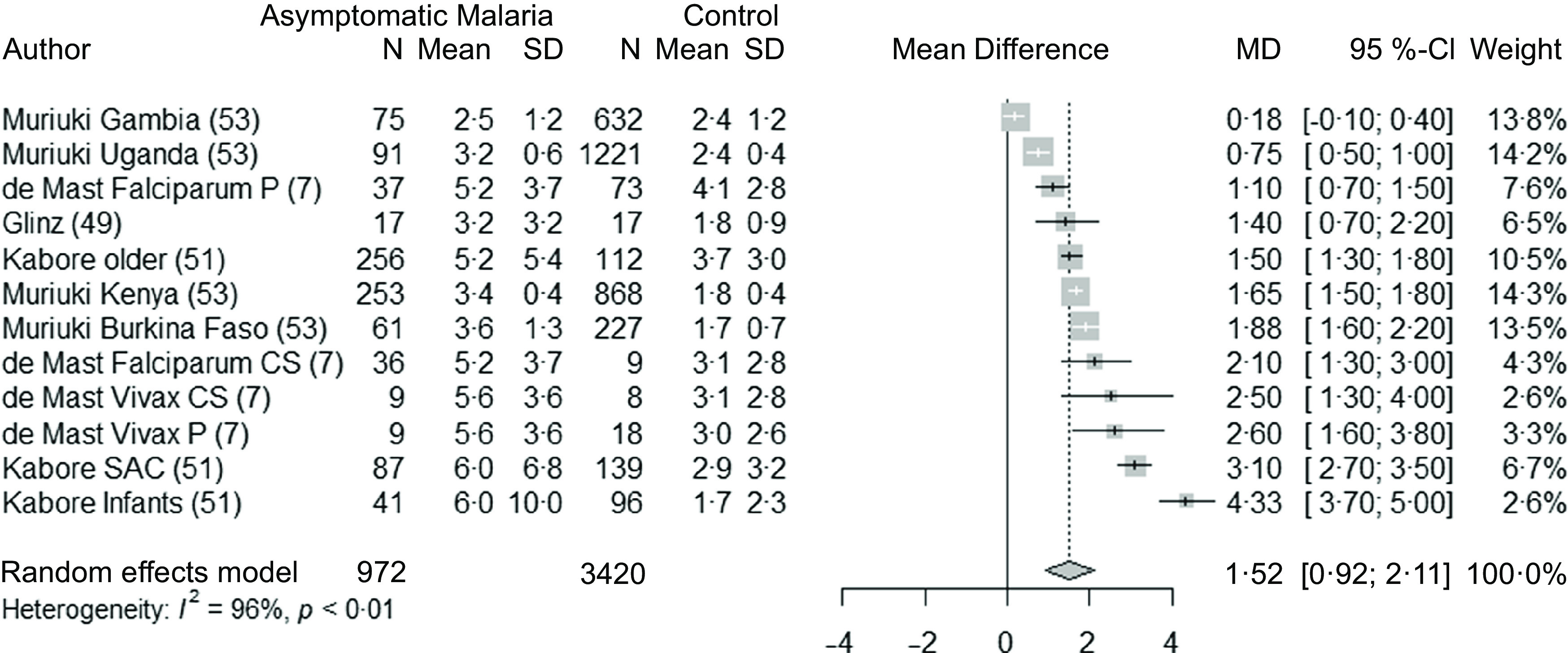
Forest plot for differences in hepcidin concentration (nmol/l) in children between malaria parasitaemia and control groups using the random effect model. The grey squares represent the mean difference from each study, while the horizontal line represents the corresponding 95 % CI. The hollow diamond represents the overall pooled effects, while the left and right points of the diamond represent the corresponding 95 % CI. SAC, school age children; P, prospective; CS, cross-sectional; MD, mean difference.

**Fig. 6.**
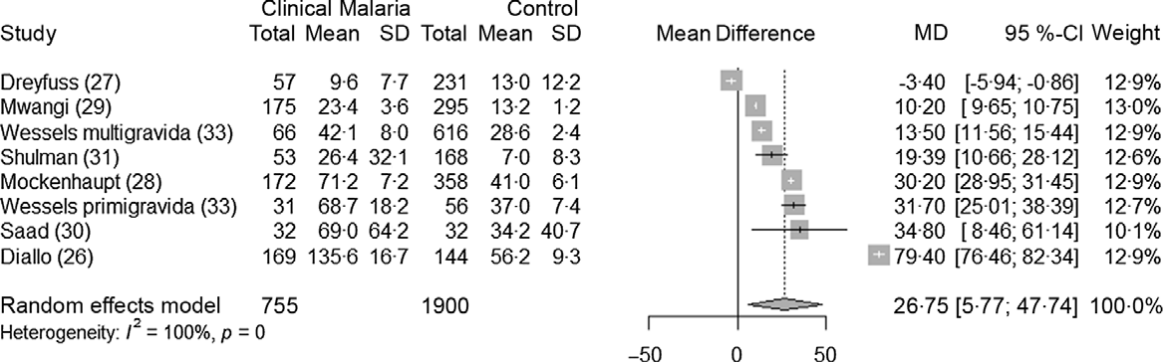
Forest plot for differences in ferritin concentrations (µg/l) in pregnant women between malaria and control group using the random effect model. The grey squares represent the mean difference from each study, while the horizontal line represents the corresponding 95 % CI. The hollow diamond represents the overall pooled effects, while the left and right points of the diamond represent the corresponding 95 % CI. MD, mean difference.

In terms of clinical malaria in adults, 269 adults were included in the analyses of 5 datasets, including an experimental infection and a study of imported cases in Europe. All the studies reported a higher ferritin concentration in the clinical malaria group (107 to 1554 µg/l elevation in the clinical group compared with the control group). In the meta-analysis, the CI was wide and included zero (493 µg/l, 95 % CI −219, 1206).

The elevation of ferritin concentrations in clinical malaria patients increased with greater severity of malaria in all studies that included this analysis, in adults and children (data not shown).

### Malaria infection and hepcidin concentrations in children and adults

Seven studies^(7,49,51,53)^ (twelve datasets) were used to analyse the association between asymptomatic malaria parasitaemia and hepcidin concentrations in children. The studies were mainly conducted in Africa apart from one study (four datasets) that was conducted in Indonesia. Hepcidin concentrations were 1·52 nmol/l (95 % CI 0·92, 2·11, *P* < 0·001) greater in malaria-infected groups compared with controls (Fig. 5). No interaction was reported in the subgroup analyses (Table 6). The heterogeneity was high (I^2^ = 96 %). The leave-one-out analysis and the sensibility analysis by risk of bias showed the stability of the pooled result (Supplementary Fig. 7 and Table 4). There was no significant publication bias, as assessed by the funnel plot and Egger’s test (Supplementary Fig. 8).

**Fig. 7.**
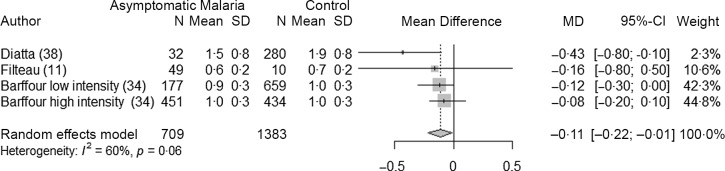
Forest plot for differences in retinol concentration (µmol/l) in children between asymptomatic malaria and control group using the random effect model. The grey squares represent the mean difference from each study, while the horizontal line represents the corresponding 95 % CI. The hollow diamond represents the overall pooled effects, while the left and right points of the diamond represent the corresponding 95 % CI. MD, mean difference.

**Fig. 8.**
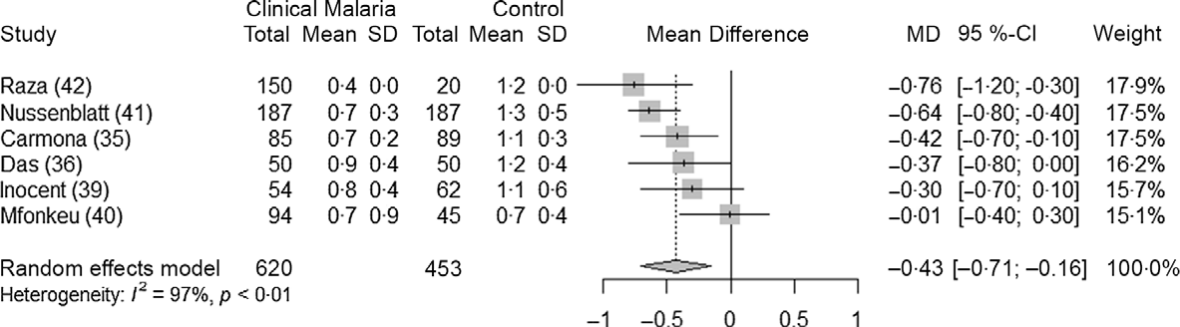
Forest plot for differences in retinol concentration (µmol/l) in children between clinical malaria and control group using the random effect model. The grey squares represent the mean difference from each study, while the horizontal line represents the corresponding 95 % CI. The hollow diamond represents the overall pooled effects, while the left and right points of the diamond represent the corresponding 95 % CI. MD, mean difference.

Two studies^(47,51)^ reported on asymptomatic malaria infection in adults and hepcidin concentrations, including 242 adults in total. There was a non-significant elevation in hepcidin in the malaria group of 0·3 nmol/l (95 % CI −10·3, 10·9). Two studies reported concentrations of hepcidin in clinical malaria infection in children^(46,51)^ and reported a non-significant elevation in hepcidin of 10·8 nmol/l (95 % CI −18·1, 39·7) in the malaria group. In adults^(48,51)^, there was also a non-significant elevation of hepcidin of 6·3 nmol/l (95 % CI −21·6, 34·3) in the malaria group.

### Malaria infection and ferritin concentrations in pregnant women

Seven studies^(26–31,33)^ analysed ferritin concentrations in pregnant women with or without malaria infection, as defined by a positive parasitaemia. The authors did not systematically report the presence of clinical symptoms. Pregnant women with malaria parasites had greater ferritin concentrations than control pregnant women without parasites (+26·8 µg/l, CI 5·8, 47·7, *P* = 0·02) (Fig. 6). The subgroup analysis revealed that the mean difference in ferritin was higher in settings with high malaria transmission (Table 7). Heterogeneity was high (I^2^ = 100 %). The sensitivity analysis showed the stability of the pooled result (Supplementary Fig. 9 and Supplementary Fig. 10). After excluding the study with high risk of bias, the difference in ferritin concentration was lower (+18·1 µg/l, 95 % CI 5·6, 30·7, *P* = 0·01) (Table 4).

**Table 6.** Results of subgroup analyses for hepcidin concentration (nmol/l) in children with asymptomatic malaria parasitaemia and control group (Mean difference and 95 % confidence interval)

Endemic profile	Number of datasets	Mean difference	95 % CI	*I* ^2^ for heterogeneity	*P*_subgroup_
Moderate intensity	6	1·86	0·62, 3·1	98 %	0·88
Low intensity	5	1·77	1·25, 2·29	68 %	
Malaria species					0·12
* P. falciparum*	10	1·77	1·04, 2·5	97 %	
* P. vivax*	2	2·56	2·46, 2·66	0 %	
Age					0·54
<60 months	6	1·95	0·74, 3·16	98 %	
>60 months	6	1·66	1·21, 2·11	60 %	
Design of the study					0·46
Cross-sectional	9	1·95	1·14, 2·76	98 %	
Prospective	3	1·58	0·73, 2·42	71 %	

**Table 7.** Results of subgroup analyses for ferritin concentration (µg/l) in pregnant women with malaria parasitaemia and control group (Mean difference and 95 % confidence interval)

Endemic profile	Number of datasets	Mean difference	95 % CI	*I* ^2^ for heterogeneity	*P*_subgroup_
High intensity	2	54·8	–258, 367	99 %	<0·001
Moderate intensity	2	22·3	–93, 138	96 %	
Low intensity	3	16·5	–7·6, 40·6	74 %	

**Fig. 9.**
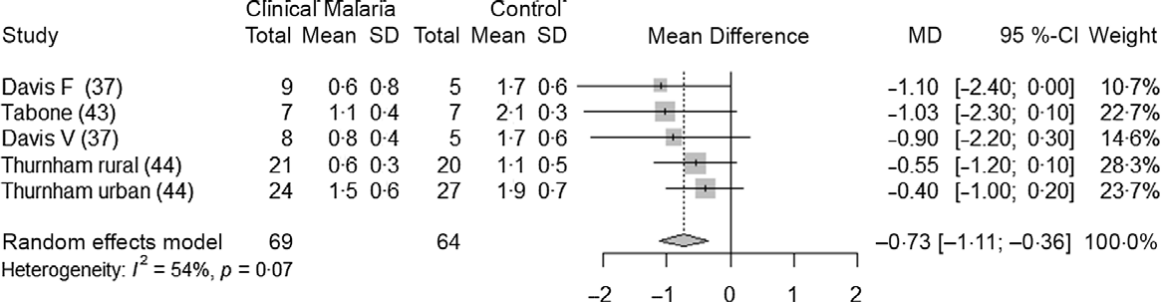
Forest plot for differences in retinol concentration (µmol/l) in adults between clinical malaria and control group using the random effect model. The grey squares represent the mean difference from each study, while the horizontal line represents the corresponding 95 % CI. The hollow diamond represents the overall pooled effects, while the left and right points of the diamond represent the corresponding 95 % CI. F, falciparum; V, vivax; MD, mean difference.

### Asymptomatic malaria infection and retinol concentrations in children and adults

Three studies^(11,34,38)^ (four datasets) analysed the association between malaria and retinol concentrations in asymptomatic children (709 children with malaria and 1383 control children). All studies were conducted in Africa and the species involved was always *P. falciparum*. Overall, retinol concentrations were lower, that is, −0·11 µmol/l (95 % CI −0·22, −0·01, *P* = 0·04) in children with asymptomatic malaria compared with control group (Fig. 7). We did not perform subgroup analyses because of the limited number of studies. The heterogeneity was moderate (I^2^ = 60 %) and the sensitivity analysis showed the stability of the pooled result after the leave-one-out analysis, and after removing the studies with high risk of bias (Supplementary Fig. 11, Supplementary Fig. 12 and Table 4).

There were no studies on the associations between asymptomatic malaria and retinol concentrations in adults.

### Clinical malaria infection and retinol concentrations in children and adults

Six studies^(35,36,39–42)^ analysed the association between clinical malaria and retinol concentrations in children (620 children with malaria parasitaemia and 453 control children). The analysis showed that retinol concentrations were reduced during an infection (−0·43 µmol/l, 95 % CI −0·71, −0·16, *P* = 0·01) (Fig. 8). When the studies with high risk of bias were excluded, the mean difference in retinol concentration was no longer statistically significant (Table 4). There were no differences noted in the subgroup analyses (Table 8).

**Table 8.** Results of subgroup analyses for retinol concentration (µmol/l) in children with clinical malaria parasitaemia and control group (Mean difference and 95 % confidence interval)

	Number of datasets	Mean Difference	95 % CI	I^2^ for heterogeneity	Psubgroup
Endemic profile					0·55
High intensity	3	–0·34	–0·70, 0·03	80 %	
Low intensity	3	–0·47	–0·68, −0·27	0 %	
Age					0·9
< 60 months	4	–0·43	–0·75, −0·1	74 %	
> 60 months	2	–0·40	–0·45, −0·35	0 %	

Three studies^(37,43,44)^ (five datasets) analysed the association between clinical malaria and retinol concentrations in adults (69 adults with malaria and 64 control adults). Two of the studies were conducted in Asia, and they were both conducted in settings with very low intensity of transmission. One was conducted in Europe with imported cases, and the author did not specify the endemicity profile of the country of origin. Retinol concentrations were lower in adults with clinical malaria compared with healthy control adults, by 0·73 µmol/l (95 % CI −1·11, −0·36, *P* = 0·005) (Fig. 9). Due to the limited number of studies, we did not perform any subgroup analyses. The heterogeneity was moderate (I^2^ = 54 %). The leave-one-out analysis showed the stability of pooled results (Supplementary Fig. 13 and Supplementary Fig. 14). Only one study in this analysis was considered at low risk of bias (Table 4).

Only one study^(32)^ reported data on retinol concentrations in pregnant women with malaria. They found a significantly reduced concentration of retinol in pregnant women with malaria (−0·54, 95 % CI −0·67, −0·41, *P* < 0·001).

### Malaria infection and retinol binding protein concentrations in children and adults

One study^(63)^ conducted in Burkina Faso with 262 children found that children with asymptomatic malaria had lower RBP values than the control group, and the mean difference was −0·13 (95 % CI −.17, −0·09, *P* < 0·001). One study^(36)^ conducted in India with 100 children presented data on RBP concentration in children with clinical malaria and found that children with clinical malaria had lower RBP values than the control group and the mean difference was −1·52 (95 % CI −1·70, −1·35, *P* < 0·001).

Only one study reported RBP data in adults during clinical malaria^(43)^, and the sample size was too small to report the data (seven patients).

## Discussion

We conducted several meta-analyses to estimate associations between malaria infection and nutrition biomarkers by using data from cross-sectional and prospective studies. Although mostly based on data from observational studies, our analyses indicate that malaria infection is associated with increased ferritin and reduced retinol concentrations even in asymptomatic infections, when individuals might not have elevated markers of inflammation.

### Association between malaria and iron indicators

The results provide strong and consistent evidence that malaria infection, asymptomatic or symptomatic, is associated with increased ferritin concentrations in children. This result was expected, as ferritin synthesis is highly upregulated by inflammatory cytokines and by infections including malaria^(53,66,67)^. In our analyses, the increase in ferritin concentration during an asymptomatic infection was similar in children and adults, and there were no differences noted in the subgroup analyses. The quality of the evidence for children seems strong as the sample size for analysis is large, the CI is relatively small and the sensitivity analysis did not reveal any significant influencer in the results or publication bias. Moreover, studies included in this analysis were considered of good quality based on the risk of bias analysis. The increase in ferritin concentration did not vary by age group. For residents in malaria-endemic areas, parasitaemia peaks in children younger than 5 years old and subsequently declines in an age-dependent manner^(68)^. In populations living under heavy exposure to malaria and frequent infection, individuals tend to develop partial immunity against the disease earlier in childhood, which may explain why children under 5 years old did not have a greater increase in ferritin than older children. However, this interpretation is limited by the fact that age groups were not particularly well defined and therefore, there might have been differences between age groups that we were not able to observe.

Overall, in the case of an asymptomatic malaria infection, the average increase in ferritin concentration is estimated at c. 28·5 µg/l, across all settings and all groups of population. Considering that the mean value of ferritin concentration in control groups was c. 25 µg/l, this indicates that the ferritin concentration in asymptomatic malaria infection was approximately doubled compared with a control group. As a comparison, based on sixteen datasets included as part of the BRINDA collaboration, Namaste and colleagues have assessed the increase in ferritin concentrations during an inflammation process, based on elevated CRP and/or AGP^(66)^; both markers are elevated in large proportions of children in low- and middle-income countries^(69)^. This definition is imperfect, as inflammation is a complex process than cannot be captured simply by the elevation of these two acute-phase proteins. However, in the absence of other widely available biomarkers of inflammation in population-based surveys, we continue to rely on a definition on inflammation based on these two markers. Namaste reported that ferritin concentration increases from 19·5 µg/l in the reference group to 50·8 µg/l during the early convalescence phase of an inflammatory episode, which in this analysis was defined as when CRP and AGP concentrations are at their highest^(66)^. This represents an increase of about 30 µg/l, which is in the same range as the increase we see during a malaria asymptomatic infection. For women of reproductive age, they report an increase in the same range (c. 30 µg/l).

The recent WHO guidelines on the use of ferritin concentrations to define iron status recommend adjusting for inflammation and indicate that it is possible to adjust for malaria. There are, however, currently no details on why this adjustment should be made or whether some specificities, such as the severity of the infection, the population group or the endemic profile should be considered. An important question for micronutrient surveys is whether elevated acute-phase proteins fully capture the effects of malaria on micronutrient markers, as might be assumed from the similar differences in ferritin due to malaria or CRP plus AGP, or whether we should account for both inflammation and malaria. Several studies included in this review report that not all children with asymptomatic malaria have elevated CRP or AGP. In the study in Burkina Faso in children with asymptomatic malaria, Barffour reported that only half of the children with malaria also had elevated AGP during low malaria season, based on AGP concentrations >1 g/l^(10)^. Righetti found similar results in Cote d’Ivoire. Among children 6–8 years old with asymptomatic malaria, 55 % had neither CRP concentration > 5 mg/l nor AGP concentration > 1g/l^(57)^. In non-pregnant women, this proportion was even higher (65 %). Similarly, de Mast found low circulating concentrations of CRP in Indonesian schoolchildren with asymptomatic parasitaemia, and 84 % of them had CRP concentrations < 5 mg/l, the threshold to define inflammation^(7)^. These findings could be attributed to the use of thresholds for CRP and AGP and might mask a mild elevation in inflammatory markers. Several studies found that even after adjusting for raised CRP and/or AGP with the regression method, ferritin concentrations were higher in children suffering from asymptomatic malaria than in the control group^(63,70)^. In children 6–23 months old in Burkina Faso, Wessells found that after adjusting for acute-phase proteins, children with asymptomatic malaria had greater plasma ferritin concentrations than the control group (23·5 ± 1·5 µg/l *v*. 11·1 ± 0·8 µg/l; *P* < 0·001)^(63)^. Muriuki found that children with malaria had greater ferritin concentrations at every decile of CRP, compared with those without malaria. Even in the lowest decile of CRP, the difference in ferritin between children with malaria and without malaria was of about 20 µg/l. They found that malaria parasitaemia also increased ferritin levels independently of increased CRP and/or AGP in multivariable analyses^(53)^. In longitudinal studies looking at ferritin concentrations and inflammatory markers concentrations after a malaria infection, it is notable that, even if CRP and AGP concentrations are slightly elevated during an asymptomatic infection, their concentrations go back to normal rapidly once the malaria infection is cleared, while ferritin concentrations remain elevated for about 1 month after the infection^(47,49)^. Considering this, we can assume that individuals with an asymptomatic malaria infection are either not suffering from inflammation or have elevated CRP and AGP for a period of time that is shorter than the time needed for their ferritin concentration to return to normal.

The highest increase in ferritin is unsurprisingly seen in clinical malaria, even though we could not reach a conclusion on clinical infection and ferritin in adults, due to the high heterogeneity and the small number of adults included. In children, the highest increase in ferritin was observed in the countries with a moderate parasite rate. In pregnancy, there was also a significant increase in ferritin concentrations in pregnant women with malaria, of c. 27 µg/l. The difference in mean concentration was greater in settings with high transmission intensity.

Hepcidin concentrations were also increased during malaria infections. As for the ferritin data, these datasets included children with and without raised CRP or AGP, and we can assume that the increase in hepcidin concentration might be occurring through both an inflammatory^(49)^ and a non-inflammatory pathway, as suggested previously^(14,53)^. Hepcidin reduces iron absorption from the gut and increases iron sequestration, resulting in a decrease of iron in the blood and a decrease in erythropoiesis^(49)^. Considering the high proportion of the population suffering from asymptomatic malaria infections in sub-Saharan Africa, this increase in hepcidin concentration could help to explain why iron deficiency prevalence remains high in population surveys and why iron supplementation and iron fortification programmes have been less effective than expected^(71)^.

### Associations between malaria and indicators of vitamin A deficiency

Children with an asymptomatic infection had lower values of serum retinol than the control group. In case of a clinical infection, the reduction was greater. In adults, lower values of serum retinol were also observed in case of a clinical infection. The reductions we observed in children and adults with a clinical infection (respectively 0·43 and 0·73 µmol/l) were greater than the reductions in retinol due to inflammation defined by elevated CRP and/or AGP, reported by the BRINDA collaboration. The BRINDA collaboration reported that inflammation reduces retinol by 0·27 µmol/l in preschool children and by 0·24 µmol/l in women of reproductive age^(66)^. However, the populations are different as BRINDA is more likely to include datasets coming from healthy participants while in these specific analyses, patients were ill and hospitalised due to malaria. Also, in these analyses, not all endemic profiles were represented. These results need to be interpreted with caution as the sample size for these analyses were small. The acute-phase response to either infection or inflammation affects retinol homoeostasis, and substantial vitamin A can be lost in the urine during illness accompanied by high fever^(3)^. There may also be increased tissue retinol uptake due to increased needs for retinol in infection which could decrease plasma retinol concentrations^(44)^. An alternative explanation is that sick children tend to eat less, and malaria could lead to vitamin A deficiency in children who already had low reserves of vitamin A^(72)^.

### Implications for large-scale surveys interpretation

The magnitude of change in ferritin and retinol concentrations in the case of an asymptomatic infection is likely to affect the estimations of iron and vitamin A deficiency in population-based surveys. Asymptomatic individuals are often present in these surveys. They are likely to experience an increase in ferritin without a significant elevation of CRP and AGP values, and therefore their ferritin values would not be adjusted by the BRINDA method as it is recommended by WHO. With regard to retinol, even if BRINDA has recommended to adjust for inflammation in children, WHO does not currently recommend any adjustment. Children and adults with asymptomatic infection could be wrongly considered as iron replete or vitamin A-deficient, and the validity of the deficiency prevalence estimates could be affected. Adjusting for malaria is only referred to as a ‘possible adjustment’ in the WHO guidelines on ferritin, and there is no mention of asymptomatic infections. However, we observed that ferritin concentrations were elevated by 28·2 µg/l in asymptomatic children and by 366 µg/l in children with clinical malaria compared with healthy children. Retinol concentrations were reduced by 0·11 µmol/l in asymptomatic children and by 0·43 µmol/l in children with clinical malaria. These data suggest an important difference according to the severity of infection, and this could have important repercussions in the assessment of iron and vitamin A status in populations where different forms of malaria infections co-exist. Even if individuals with clinical malaria are not likely to be included in surveys, people recovering from clinical malaria might be. Applying a single correction factor to all forms of malaria, as is currently recommended in the WHO guidelines, would certainly affect estimates coming from micronutrient surveys and other surveys that assess the iron status of a sample of a population in a malaria endemic setting. More research should be done to confirm whether the study setting should be considered when applying an adjustment, as our data seem to indicate that a clinical infection could have different repercussions on ferritin concentrations depending on the endemic profile. We did not have enough datasets to analyse infections with *P. vivax*, and it requires further research. In our analyses, the use of different malaria diagnostic methods did not seem to impact the magnitude of the effects.

### Limitations

The primary limitation to these analyses is the variability between studies, including the large age range among children in some of the datasets. Another limitation is the population used as a control group, as most studies had different strategies to recruit their control group. We did not investigate whether the children were suffering from hookworm or Schistosoma infections, which could have affected further their iron status. Another limitation is the generalisability of the results related to clinical infections. Most of the studies including clinical infections were conducted at the hospital, whereas in population-based surveys conducted to measure the micronutrient status of a population, participants would be sampled in the community. We also acknowledge that our search strategy, despite being large and inclusive of three languages of publication, did not include grey literature or regional databases, which might have introduced a selection bias in the systematic review. Finally, although there were many studies on the association of ferritin and malaria in children, fewer studies were included in other meta-analyses, and these included studies had higher risk of bias, suggesting caution is required in interpreting these results.

### Conclusion

The findings of this systematic review and meta-analysis suggest that malaria infection should be measured and adjusted for in nutritional surveys of populations living in malaria endemic areas, particularly for assessments of iron status. Malaria test results should be reported in population-based surveys, as well as a measure of clinical symptoms in the participant. This will allow more accurate adjustment of serum ferritin concentrations to define individual iron status. Preliminary analyses indicate that retinol concentration also is affected by malaria, but not enough data are currently available to support firm conclusions for children and adults. Further research is needed to develop individualised adjustment methods that can take into account the concentrations of acute-phase proteins and the presence, and severity, of a malaria infection.

## Supplementary material

For supplementary material accompanying this paper visit https://doi.org/10.1017/S0007114522000757. click here to view supplementary material
