# Supplementary material for: Measuring the impact of malaria infection on indicators of iron and vitamin A status: a systematic literature review and meta-analysis
Source: Br J Nutr. 2022 Mar 9;129(1):87–103. doi: 10.1017/S0007114522000757 (PMC9816655; doi:10.1017/S0007114522000757)
Supplement: Supplementary file 1 [file S0007114522000757sup.zip › S0007114522000757sup001.pdf]

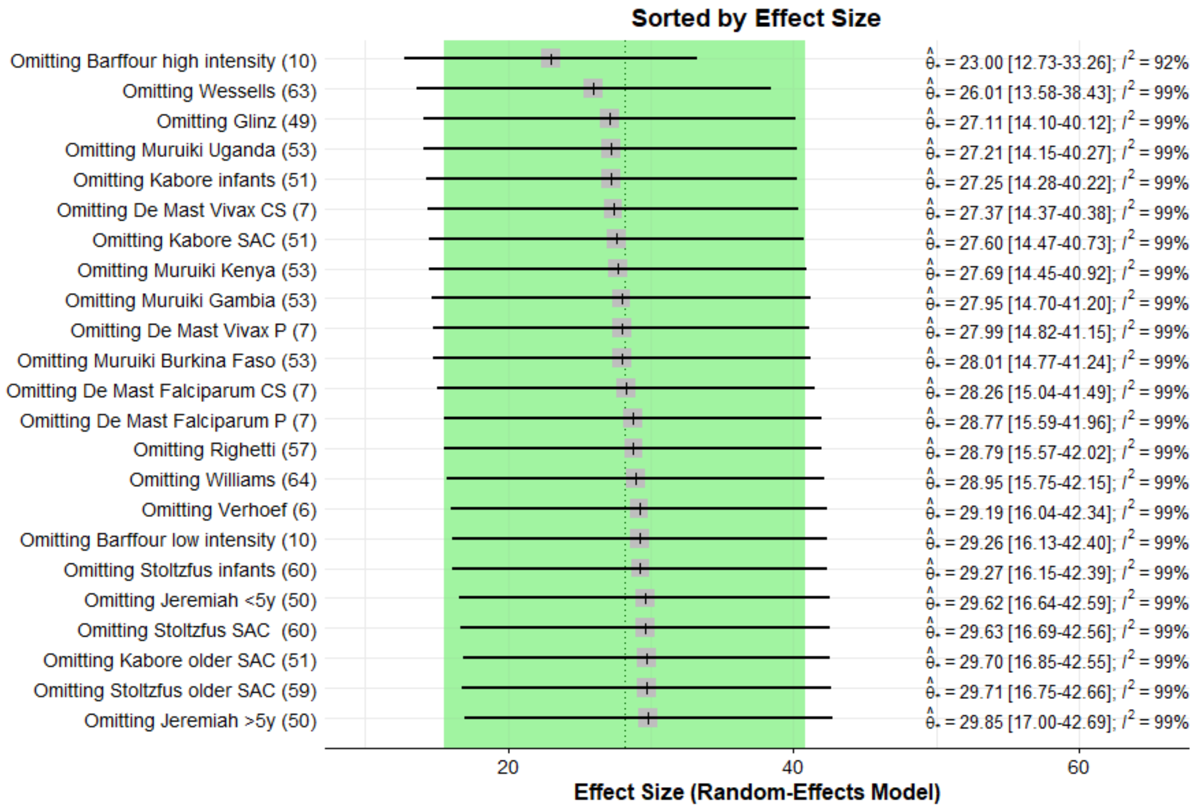

Supplementary figure 1: Sensitivity analysis of ferritin concentration (µg/L) between asymptomatic malaria and control in children, using leaving-one out analysis. Recalculated pooled effects, with one study omitted each time. The dashed line and shaded area represent the original pooled mean difference and its 95% confidence interval. CS, cross-sectional; SAC, school-age children; P, prospective

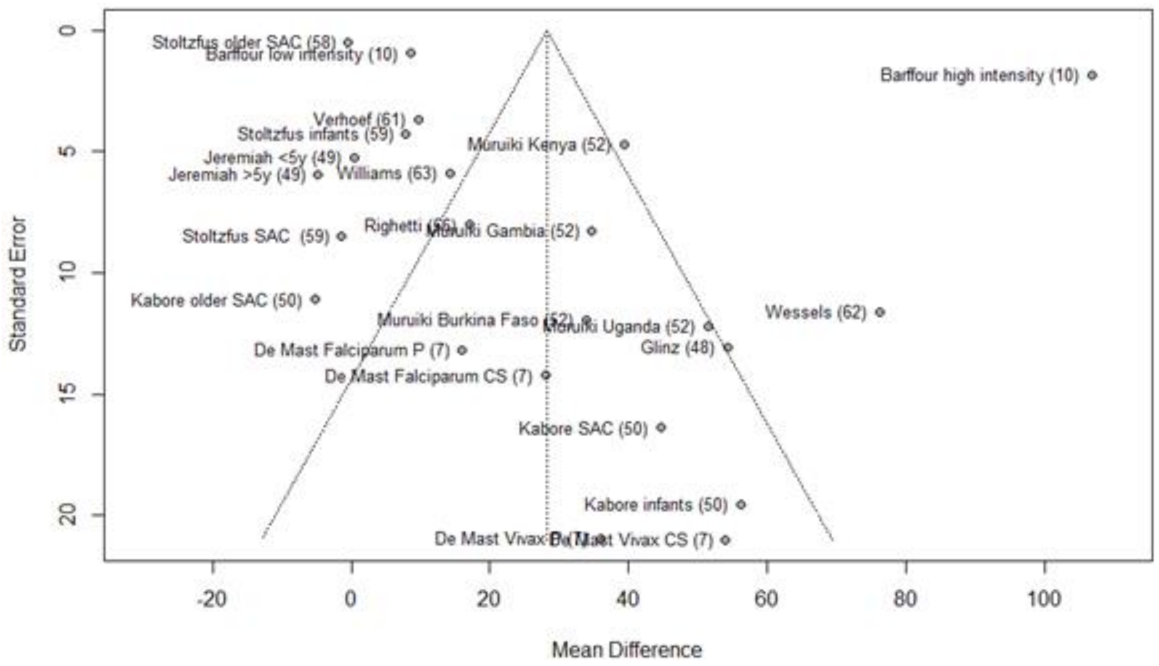

Supplementary figure 2: Funnel plot showing publications related to asymptomatic malaria and ferritin concentration (µg/L) in children. CS, cross-sectional; SAC, school-age children; P, prospective

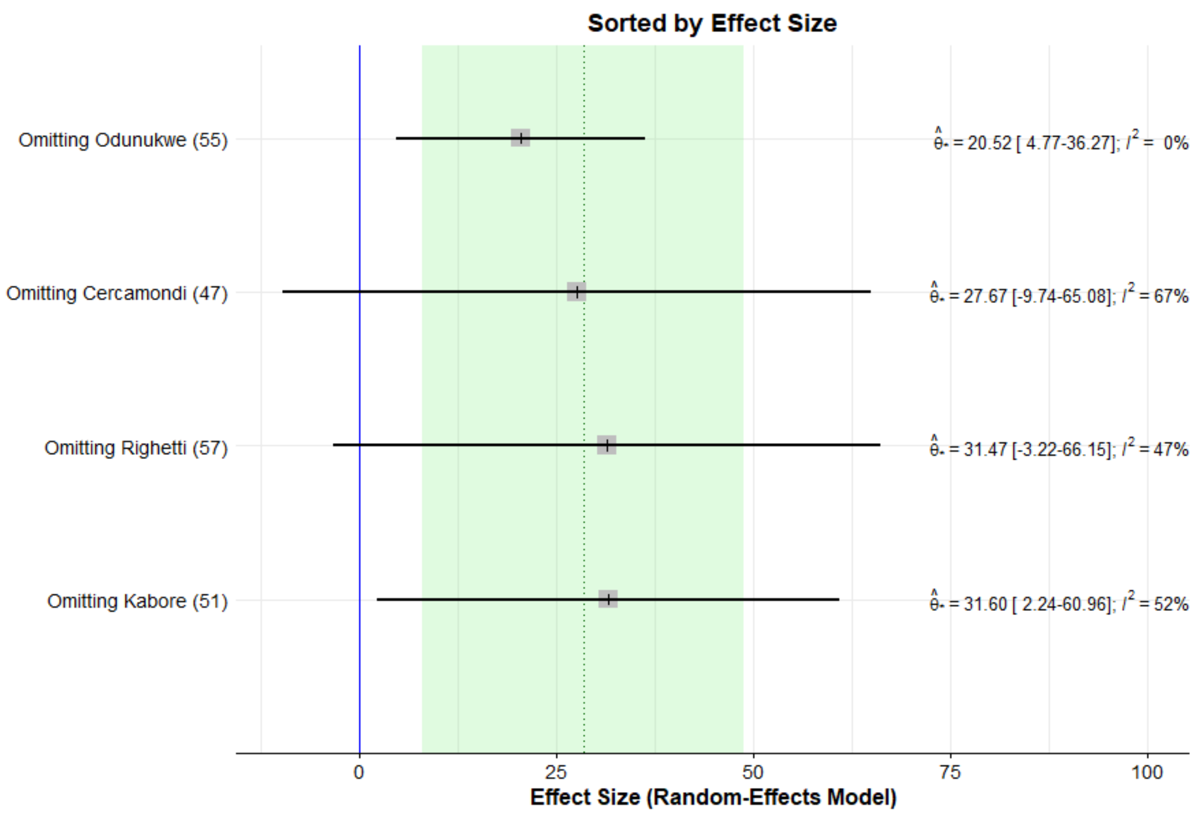

Supplementary figure 3: Sensitivity analysis of ferritin concentration (µg/L) between asymptomatic malaria and control in adults, using leaving-one out analysis. Recalculated pooled effects, with one study omitted each time. The dashed line and shaded area represent the original pooled mean difference and its 95% confidence interval.

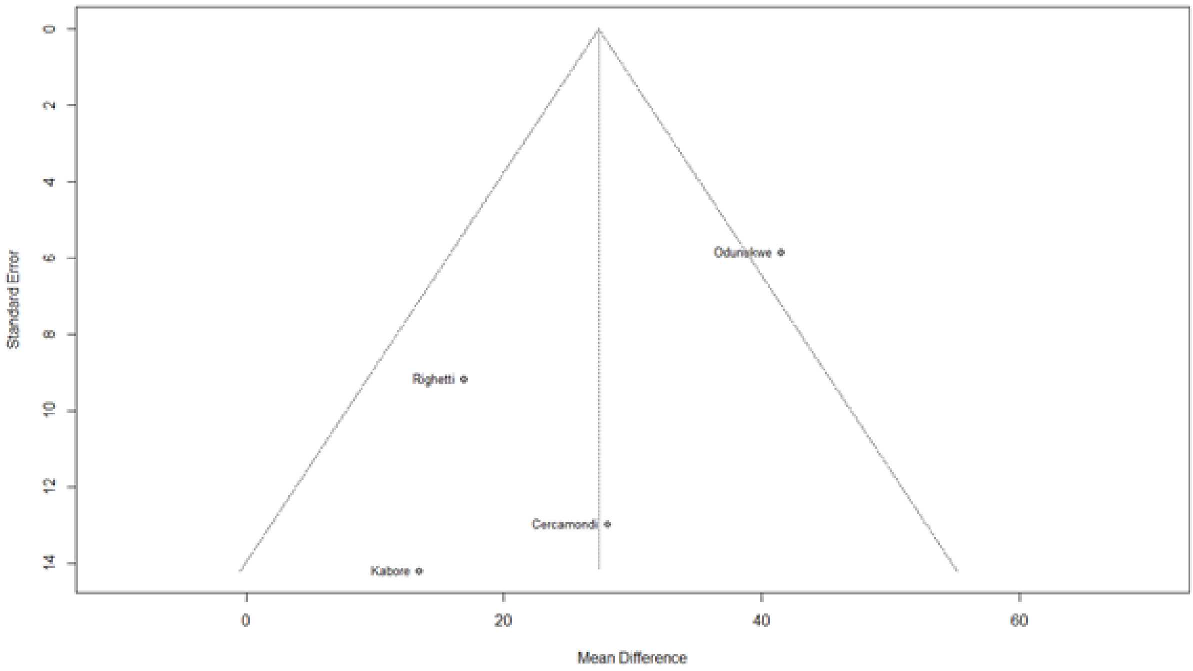

Supplementary figure 4: Funnel plot showing publications related to asymptomatic malaria and ferritin concentration (µg/L) in adults

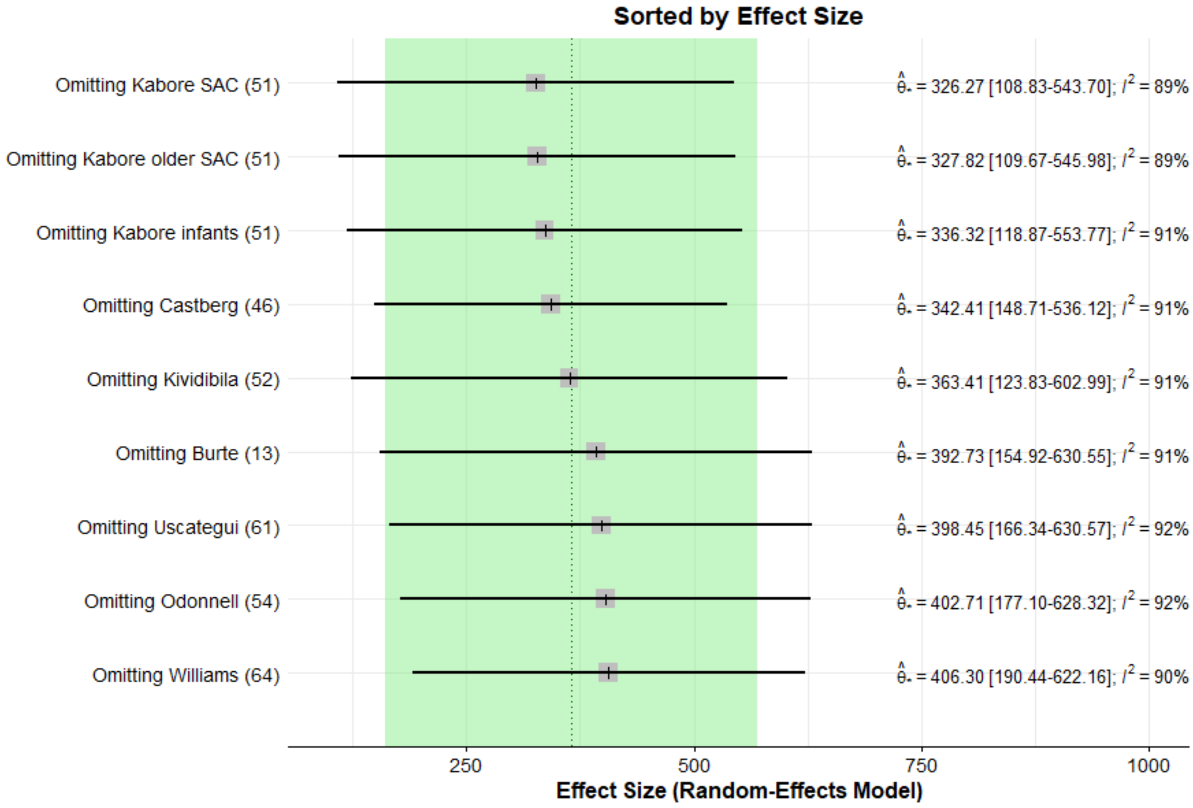

Supplementary figure 5: Sensitivity analysis of ferritin concentration (µg/L) between clinical malaria and control in children, using leaving-one out analysis. Recalculated pooled effects, with one study omitted each time. The dashed line and shaded area represent the original pooled mean difference and its 95% confidence interval. SAC, school-age children

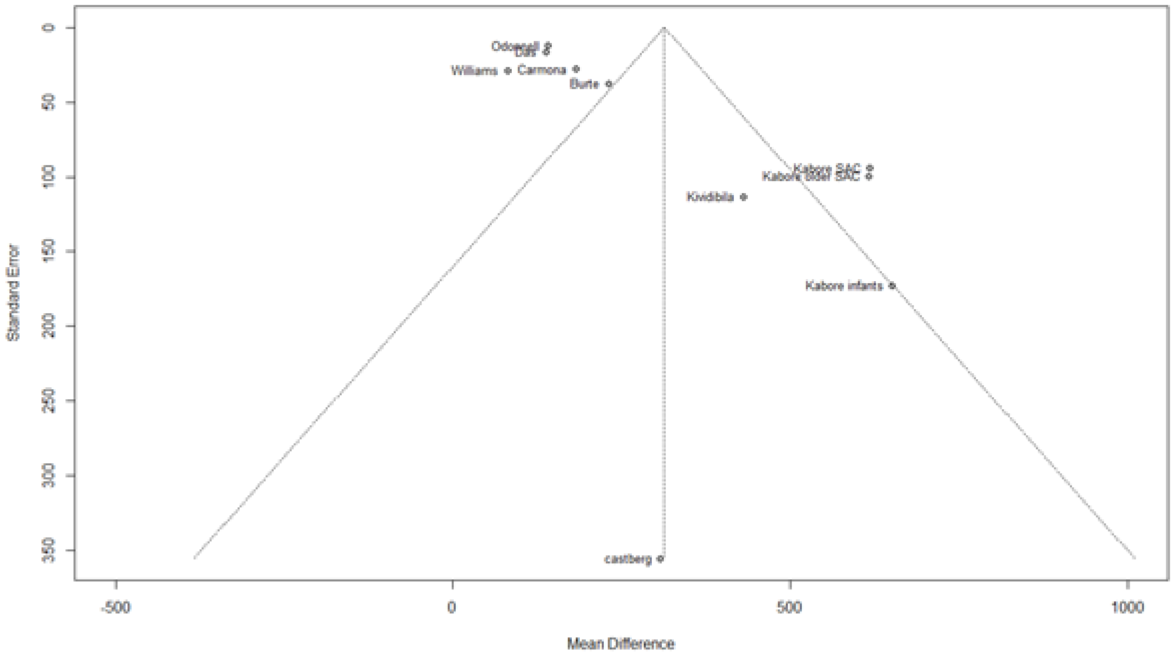

Supplementary figure 6: Funnel plot showing publications related to clinical malaria and ferritin concentration (µg/L) in children. SAC, school-age children

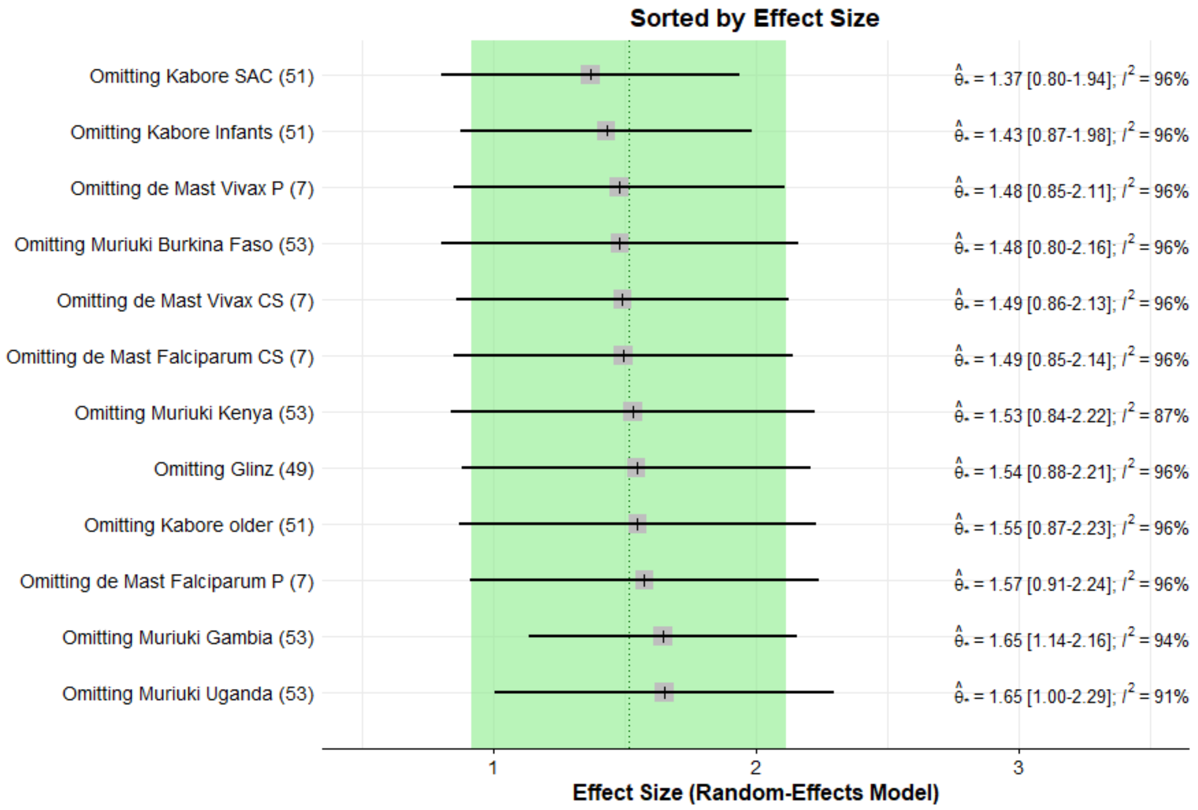

Supplementary figure 7: Sensitivity analysis of hepcidin concentration (nmol/L) between asymptomatic malaria and control in children, using leaving-one out analysis. Recalculated pooled effects, with one study omitted each time. The dashed line and shaded area represent the original pooled mean difference and its 95% confidence interval. SAC, school-age children; P, prospective; CS, cross-sectional.

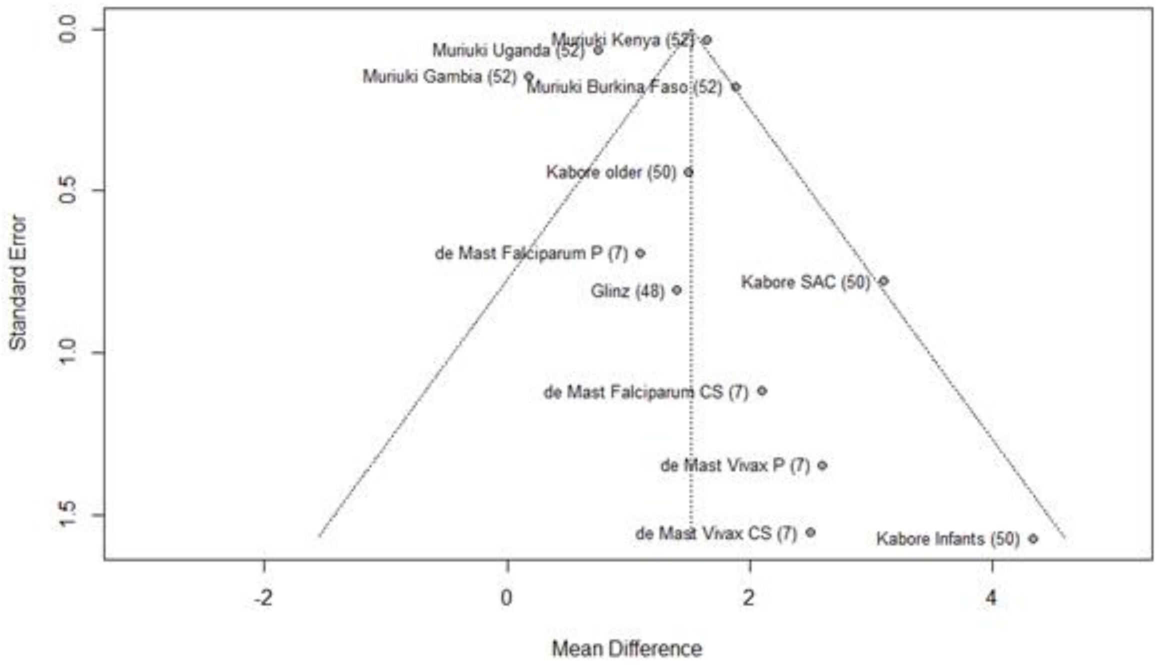

Supplementary figure 8: Funnel plot showing publications related to asymptomatic malaria and hepcidin concentration (nmol/L) in children. SAC, school-age children; P, prospective; CS, cross-sectional.

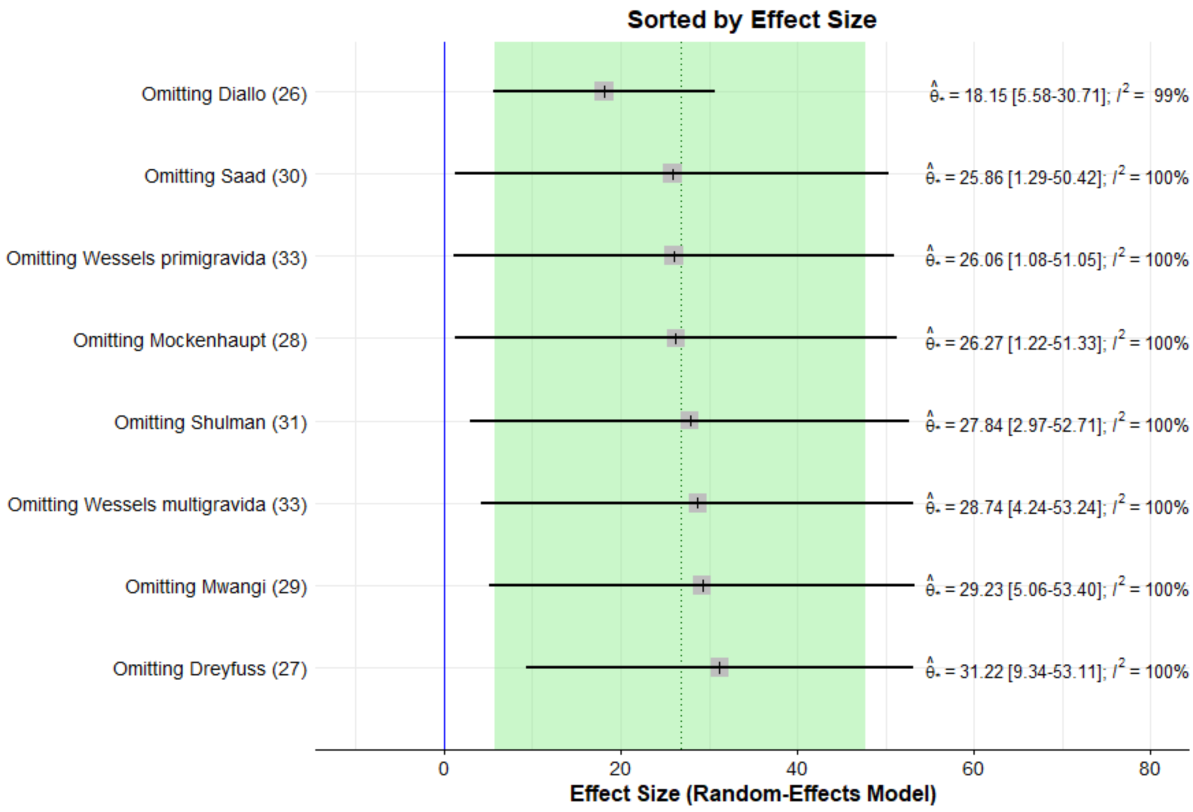

Supplementary figure 9: Sensitivity analysis of ferritin concentration (µg/L) between malaria and control in pregnant women, using leaving-one out analysis. Recalculated pooled effects, with one study omitted each time. The dashed line and shaded area represent the original pooled mean difference and its 95% confidence interval.

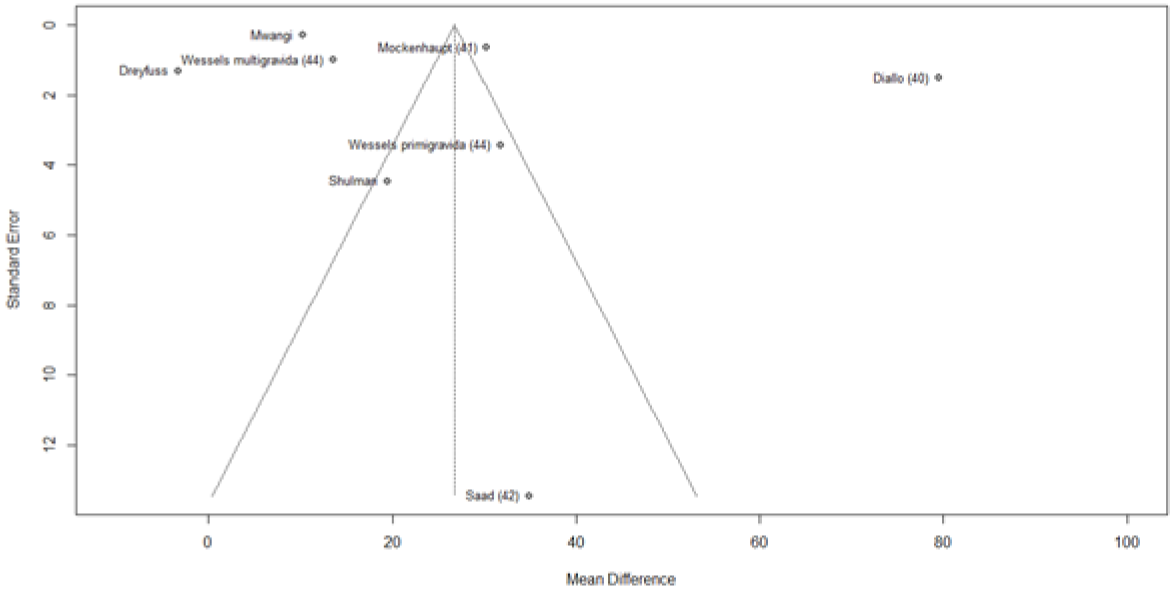

Supplementary figure 10: Funnel plot showing publications related to malaria and ferritin concentration (µg/L) in pregnant women

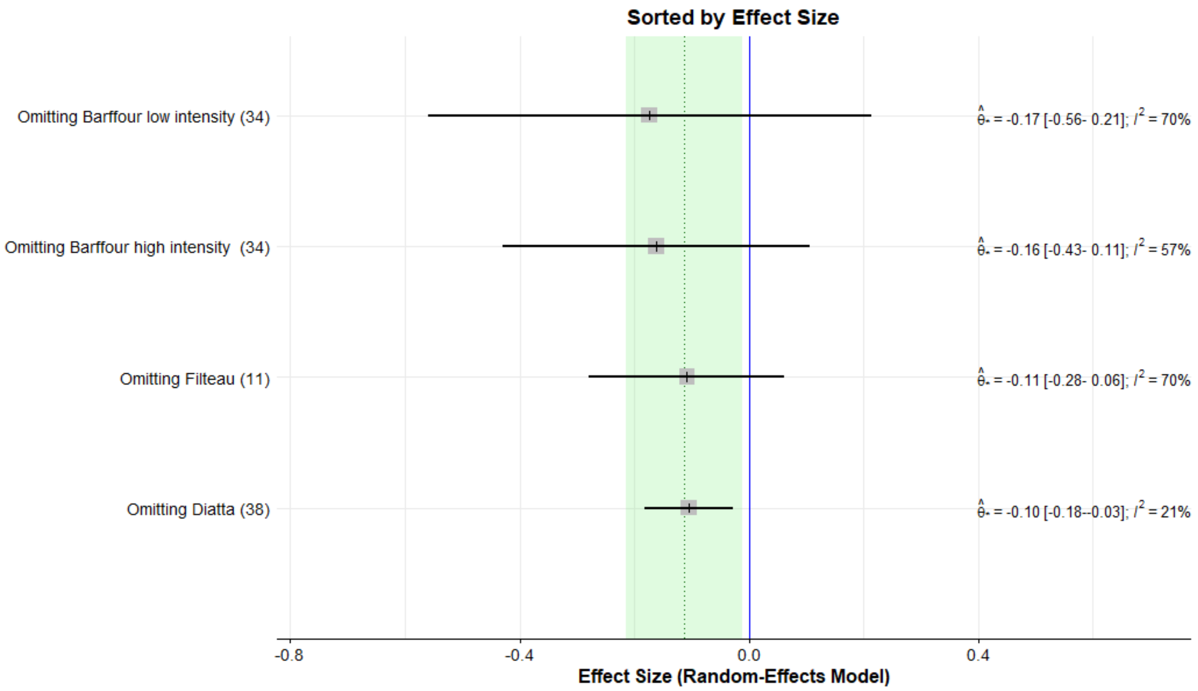

Supplementary figure 11: Sensitivity analysis of retinol concentration (µmol/L) between asymptomatic malaria and control in children, using leaving-one out analysis. Recalculated pooled effects, with one study omitted each time. The dashed line and shaded area represent the original pooled mean difference and its 95% confidence interval.

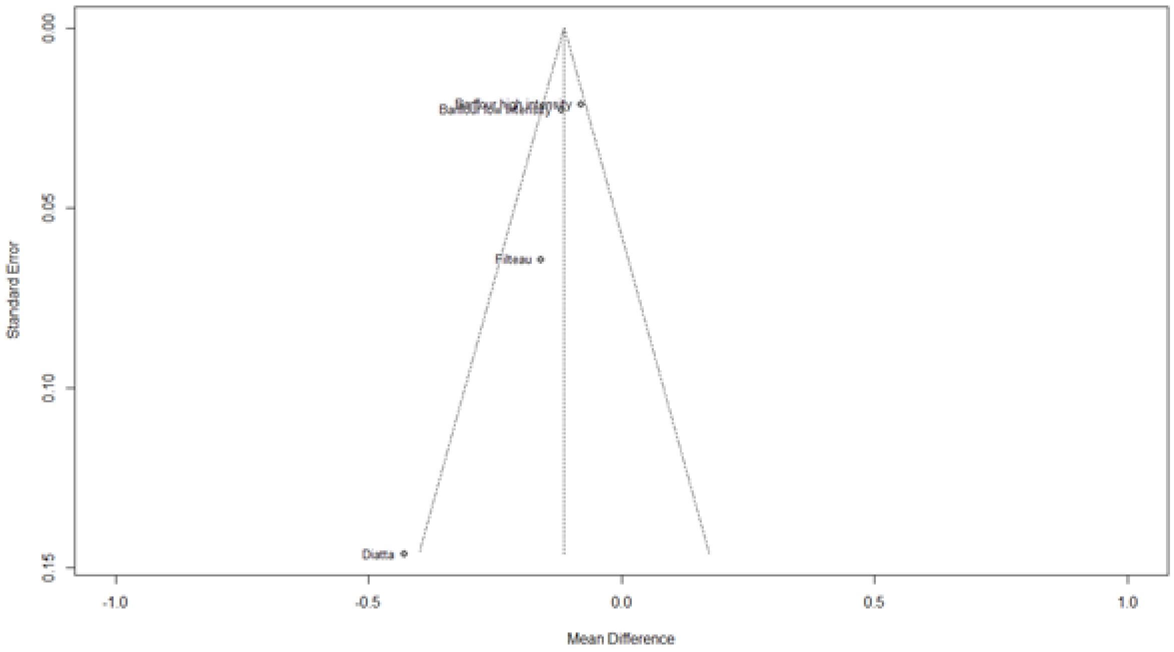

Supplementary figure 12: Funnel plot showing publications related to asymptomatic malaria and retinol concentration (µmol/L) in children

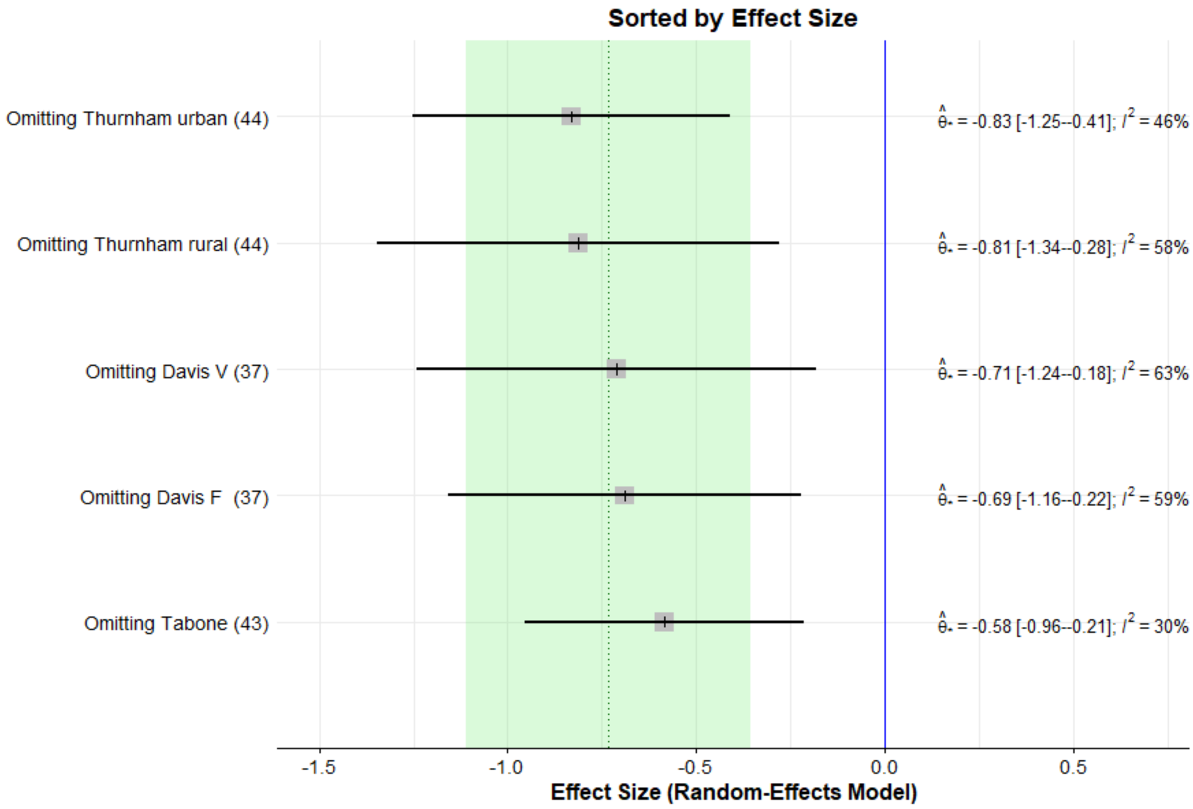

Supplementary figure 13: Sensitivity analysis of retinol concentration (µmol/L) between malaria and control in adults, using leaving-one out analysis. Recalculated pooled effects, with one study omitted each time. The dashed line and shaded area represent the original pooled mean difference and its 95% confidence interval. V, Vivax; F, Falciparum

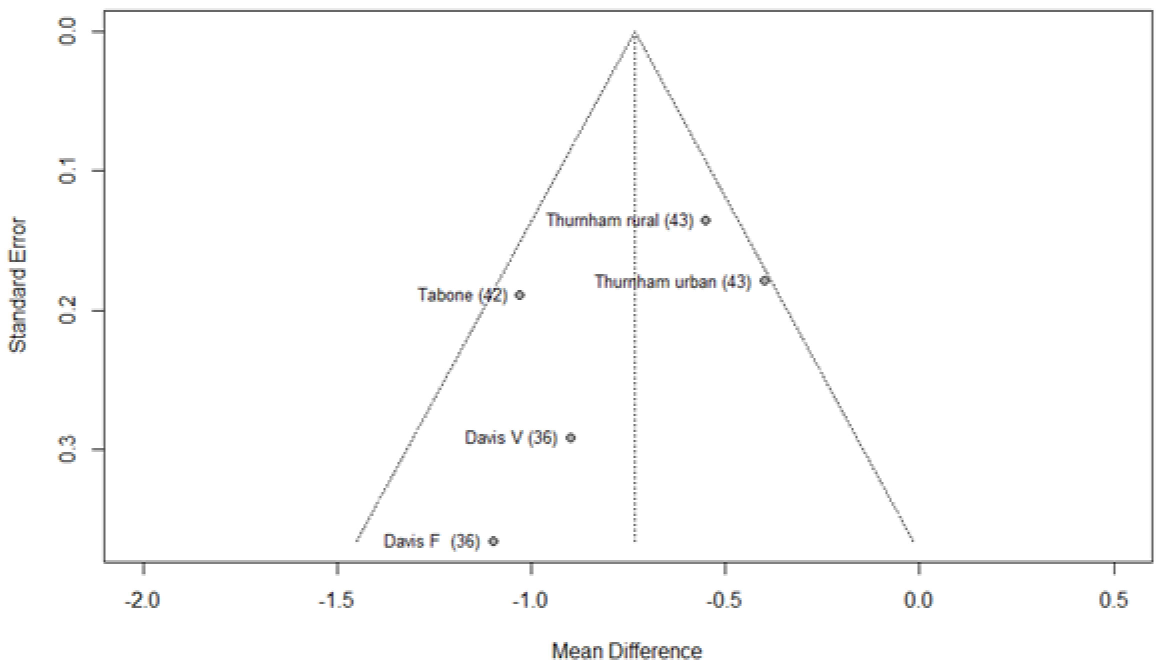

Supplementary figure 14: Funnel plot showing publications related to malaria and retinol concentration (µmol/L) in adults. V, Vivax; F, Falciparum
