# Supplementary material for: Measuring the impact of malaria infection on indicators of iron and vitamin A status: a systematic literature review and meta-analysis
Source: Br J Nutr. 2022 Mar 9;129(1):87–103. doi: 10.1017/S0007114522000757 (PMC9816655; doi:10.1017/S0007114522000757)
Supplement: Supplementary file 1 [file S0007114522000757sup.zip › S0007114522000757sup002.docx]

**Supplementary file 1: search strategy**

1. malaria.mp. or exp Malaria Vaccines/ or exp Malaria, Falciparum/ or exp Malaria, Cerebral/ or exp Malaria, Vivax/

2. anophele.mp. or exp Anopheles/

3. exp Ferritins/ or ferritin*.mp.

4. ('iron indicator' or 'iron biomarker' or 'iron absorption' or 'iron deficiency').mp. [mp=title, abstract, original title, name of substance word, subject heading word, floating sub-heading word, keyword heading word, organism supplementary concept word, protocol supplementary concept word, rare disease supplementary concept word, unique identifier, synonyms]

5. exp Retinol-Binding Proteins/ or 'retinol binding protein*'.mp. or exp Vitamin A/

6. Plasmodium.mp. or Plasmodium/

7. 1 or 2 or 6

8. exp Hepcidins/

9. ("serum retinol" or "plasma retinol").mp. [mp=title, abstract, original title, name of substance word, subject heading word, floating sub-heading word, keyword heading word, organism supplementary concept word, protocol supplementary concept word, rare disease supplementary concept word, unique identifier, synonyms]

10. 3 or 4 or 5 or 8 or 9

11. 7 and 10

Filter: human studies, published in English, French and Spanish. There is no time or geographical limitation to the search.
